# HNO Dimerization as a Chemical Reference Standard for N_2_O Isotopomer Ratio: Ab Initio Calculations, Formation Kinetics, and Frequency Comb Spectroscopy

**DOI:** 10.1021/jacs.5c09983

**Published:** 2025-10-10

**Authors:** Ibrahim Sadiek, Adrian Hjältén, Gernot Friedrichs, Aleksandra Foltynowicz

**Affiliations:** † Department of Physics, 8075 Umeå University, 901 87 Umeå, Sweden; ‡ Experimental Physics V, Faculty of Physics and Astronomy, 153606 Ruhr University Bochum, 44780 Bochum, Germany; § Institute of Physical Chemistry, 9179 Christian-Albrechts-University Kiel, 24118 Kiel, Germany; ∥ Kiel Marine Science-Centre for Interdisciplinary Marine Sciences, 9179 Christian-Albrechts-University Kiel, 24098 Kiel, Germany

## Abstract

The ^15^N-site preference of N_2_O, δ^15^N^SP^ = [^14^N^15^NO]/[^15^N^14^NO] – 1 ≈ δ^15^N^α^ – δ^15^N^β^ = SP, quantifies the relative enrichment of ^15^N at the central (α) versus terminal (β) position in nitrous oxide and serves as a robust isotopic fingerprint for tracing N_2_O sources and formation pathways. One such pathway involves dimerization of nitroxyl (HNO), which can occur directly or enzymatically. The direct dimerization of HNO in aqueous solution has been suggested to proceed via acid-base equilibria, forming *cis*-hyponitrite or *cis*-hyponitrous acid intermediates that decompose to N_2_O. Measuring δ^15^N^SP^(N_2_O) from HNO dimerization would not only test the postulated formation pathway but also offer an easily reproducible chemical reference standard for isotopic studies. Using high-precision mid-infrared frequency comb spectroscopy, we determine the ^15^N-site preference by analyzing the absorption ratio of multiple rovibrational line pairs of the α and β isotopomers. At pH = 0.62, δ^15^N^SP^(N_2_O) decreases with synthesis temperature from 36.6‰ at *T* = 278 K to 23.4‰ at *T* = 336 K, in very good agreement with prediction based on a kinetic equilibrium model of the strongly pH-dependent *cis*-hyponitrous acid/*cis*-hyponitrite acid-base system. These results confirm N_2_O formation via the *cis* pathway is dominated—at the low synthesis pH—by the decomposition of the neutral *cis*-hyponitrous acid. Alternative formation of N_2_O from *trans*-hyponitrite, predicted to yield δ^15^N^SP^ ≈ −7‰, can be excluded. Our work, combining high-precision spectroscopic measurements with first-principles ab initio and transition state theory calculations, is the first step toward establishing the chemical synthesis of N_2_O from HNO dimerization under acidic conditions as an absolute δ^15^N^SP^ reference.

## Graphical abstract

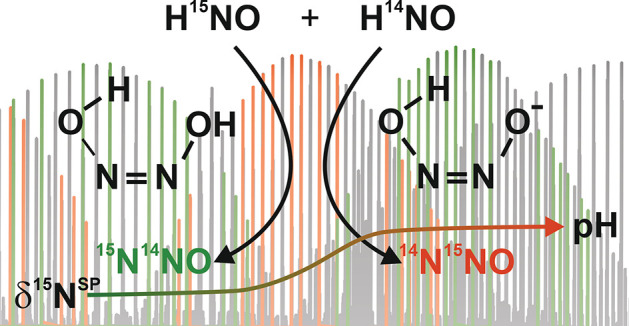

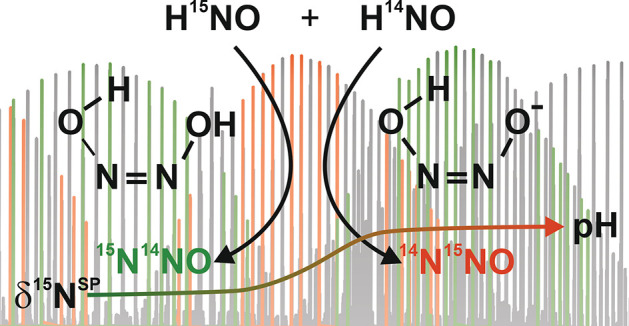

## 1 Introduction

Nitrous oxide, N_2_O, is a tracer of the nitrogen cycle, a potent long-lived greenhouse gas, and a major precursor for stratospheric ozone depletion.^1,2^ The measurement of its isotopic signatures and isotopomer ratios is a valuable tool for tracking its sources and formation mechanisms.^3^ In particular, the measurement of ^15^N-site preference, discerning the predominance of ^15^N substitution in the central, α, position over the terminal, β, position, allows for better constraining biogeochemical models for nitrogen processing in terrestrial ecosystems. This is because the site preference depends only on the mechanisms and pathways of N_2_O formation and not on the isotopic signature of the substrate. The site preference in terms of a “ratio-based” definition can be expressed in δ-notation as δ^15^N^SP^ = ([^14^N^15^NO]/[^15^N^14^NO] – 1) × 1000‰. Note that throughout the paper we use the common parts per thousand (‰) notation because the resulting isotope effects and site preferences are all small and in the range of ‰. δ^15^N^SP^ is closely related to the ‘δ- or reference-based’ definition SP = δ^15^N^α^ – δ^15^N^β^, which is the preferred measure for site preference in isotope-ratio mass spectrometry (IRMS) and is widely reported within the field of environmental science and geoscience. The SP value is anchored to the Air-N_2_ δ^15^N reference scale^4^ according to δ^15^ N^α,β^ = (*R*_α,β_/*R*_reference_ – 1) × 1000‰ with *R*_α_ = [^14^N^15^NO]/[^14^N^14^NO] and *R*_β_ = [^15^N^14^NO]/[^14^N^14^NO], respectively. In the context of our combined spectroscopic and theoretical approach, reporting the ratio-based instead of the δ-based site preference holds several advantages: (i) it can be directly linked to predictions based on reaction kinetic models and/or theoretical ab initio calculations for a sequence of (equilibrium) reactions, each of them contributing to a finally observed [^14^N^15^NO]/[^15^N^14^NO] ratio, (ii) it is independent of the overall ^15^N enrichment of the sample (note that [^14^N^14^NO] does not occur in the definition of δ^15^N^SP^), and (iii) it is intrinsically independent of any reference compound. While both expressions converge when *R*_α,β_/*R*_reference_ ratios approximate unity, potentially significant differences should be carefully accounted for when directly comparing δ^15^N^SP^ and SP values. Straightforward conversion between the two scales is possible,

$$\delta^{15}N^{\mathrm{SP}}=\frac{\mathrm{SP}}{1+\delta^{15}N^{\beta }/1000‰}$$ 1

showing that values of SP = 50‰ (or 10‰) and δ^15^N^β^ = 50‰ (10‰) result in a significant offset of δ^15^N^SP^ – SP = −2.4‰ (−0.1‰) between the two scales. Further information about the used terminology and the mathematical derivation of the mentioned SP to δ^15^N^SP^ conversion is provided in Section A of the Supporting Information.

Despite large efforts to improve interlaboratory compatibility^5^ and to consistently scale the ^15^N-site preference measurements to the international standard for the ^15^N/^14^N isotope ratio of Air-N_2_,^4^ significant uncertainties remain. Typically, the measurements are performed using either IRMS or quantum cascade laser-based absorption spectroscopy (QCLAS),^5^ where most of the calibration schemes can be traced back to the original work of Toyoda and Yoshida.^6^ Here, the thermal decomposition is isotopically characterized with the α- and β-nitrogen originating from the ${\mathrm{NO}}_{3}^{-}$ and ${\mathrm{NH}}_{4}^{+}$ ions, respectively. The challenging mass-spectrometric measurements, which require a correction for isotopic scrambling of the analyzed signal ratios of the nitrous oxide parent (N_2_O^+^) and NO^+^ fragment ion,^7^ have been independently confirmed by Fourier transform infrared spectroscopy (FTIR) measurements by Griffith et al.^8^ Nevertheless, a recent interlaboratory comparison^9^ and a detailed reassessment of combined IRMS and QCLAS study on the isotopic analysis of N_2_O reference materials for use as a community standard^10^ state an overall uncertainty of >1.5‰ for SP, presumably due to nonquantitative NH_4_NO_3_ decomposition and ion-specific isotopic enrichment factors of the generated N_2_O calibration gases. From both an analytical and mechanistic perspective, the availability of a reference material with exactly known [^14^N^15^NO]/[^15^N^14^NO] ratio is advantageous, as it not only provides a reliable reference standard for evaluating site preference measurements but also facilitates direct comparison between these measurements and theoretically predicted isotopomer ratios. In this context and as introduced by Magyar et al.,^11^ thermally (fully) equilibrated N_2_O samples may serve as such an absolute reference standard because the measured ratio [^14^N^15^NO]/[^15^N^14^NO] can be identified with the thermodynamic equilibrium constant *K*_α/β_. Experimentally, thermal equilibration can be accomplished by using metal catalysts at a moderate temperature of 200 °C, where N_2_O disproportionation (that may result in a kinetically biased [^14^N^15^NO]/[^15^N^14^NO] ratio) is still negligible. Provided that accurate spectroscopically measured or theoretically predicted ab initio data (anharmonic vibrational frequencies and moments of inertia) are available, *K*_α/β_ can be reliably calculated using statistical thermodynamics.^12^ Kantnerova et al.^13^ have already demonstrated the use of thermally equilibrated N_2_O samples as a working standard gas to develop a new calibration scheme for N_2_O clumped isotopes. Along those lines and exploring an alternative approach, in this work we exploit the dimerization reaction of HNO which—irrespective of the isotopic composition of the starting material—provides a specific [^14^N^15^NO]/[^15^N^14^NO] ratio that can be related to theoretically derived values from ab initio calculations of isotope effects and as such could serve as an absolute chemical reference standard as well.

Despite the broad use of the ^15^N-site preference for source characterization, the molecular mechanisms underlying the resulting isotopomer ratios in N_2_O are not well understood. Various biological sources exhibit characteristic SP values, ranging from SP ≈ −5‰ for N_2_O from denitrifying bacteria,^14,15^ SP ≈ 0‰ from nitrifier-denitrification,^14^ SP ≈ 30‰ for N_2_O from archaeal and bacterial nitrifiers^16,17^ to SP ≈ +37‰ for denitrifying fungi.^18^ At this point, we refer the interested reader to a very detailed compilation of previous measurements given in a recent review by Toyoda et al.^19^ In enzyme-catalyzed reactions, the chemical formation of N_2_O is suggested to take place through nitric oxide (NO) dimerization via nitric oxide reductase (NOR) enzymes found in nitrifying and denitrifying bacteria and fungi. The structures of these NOR enzymes contain iron, which is believed to act as an active center for the N–N bond formation (first dimerization step) and subsequent N–O bond cleavage.^20^ Here, the ^15^N-site preference has often been interpreted as isotopic discrimination by an intermediate hyponitrite species in metal-bridged complexes in the enzyme.^21,22^ In addition to the enzymatic pathway, the formation of N_2_O had also been suggested to originate from the dimerization of free nitroxyl (HNO, azanone), which forms after the enzymatic reduction of NO through the reaction sequence:^23^

$$\mathrm{HNO}+\mathrm{HNO}\to \left[cis‐\mathrm{HONNOH}\right]\to N_{2}O+H_{2}O$$ 2

Here, depending on the pH value, *cis*-HONNOH represents unstable *cis*-hyponitrous acid intermediates in their protonated or deprotonated forms. For such nonenzymatically driven pathways, the isotopic site preferences represent an intrinsic isotopic effect of the formed free (protonated) hyponitrite intermediates and hence should be independent of the HNO-forming substrate. For example, Toyoda et al.^15^ measured the same site preferences (SP ≈ +30‰) for hydroxylamine oxidation and nitrite reduction, where both reactions produce N_2_O through HNO dimerization.

Stimulated by the importance of HNO in biology and medicine^24–27^ as well as environmental science,^1–3^ several experimental and theoretical studies have been performed to elucidate the HNO dimerization mechanism.^28–34^ It was initially postulated that the measured ^15^N-site preference of ≈ +30‰ by Toyoda et al.^15^ can be traced back to the formation of symmetric intermediates, such as N_2_O_2_^2–^, followed by a heavy atom isotope effect in the N–O bond cleaveage.^15,35,36^ However, ab initio calculations by Fehling and Friedrichs showed that consecutive acid-base equilibria control the overall HNO dimerization mechanism.^31^ According to Fehling and Friedrichs,^31^ rapid proton transfer reactions between solute and solvent take place after the initial dimerization step, with the kinetically isolated *cis* pathways favored in the solution phase. Based on a Kirkwood model that accounts for solute–solvent dipole–dipole interaction, they estimated a preferential *cis* dimer formation with a rate constant about 500 times larger than for the *trans* dimer formation.^31^ This directly contrasts the 3-fold preference for the *trans* isomer seen in the gas phase^37^ and can be attributed to a significant solvent effect that aligns with the enhanced stabilization of the *cis* isomer’s dipole moment. Furthermore, Fehling and Friedrichs suggested that N_2_O formation is fully dominated by the decomposition of the nonsymmetric *cis*-hyponitrite anion (*cis*-HONNO^–^) at pH ≥ 1, due to an estimated low acid constant of *cis*-hyponitrous acid (p*K*_a_ = 3.1) and a much faster decomposition of *cis*-HONNO^–^ compared to the neutral *cis*-hyponitrous acid intermediate (*cis*-HONNOH). Subsequent quantum mechanics/molecular dynamics (QM/MD) simulations of Bringas et al.^32^ as well as Zhang and Thynell^33^ supported the fast acid-base mechanism as well as the preferential formation of N_2_O through the *cis* pathway. However, the revised determination of the acid constant of *cis*-hyponitrous acid by Zhang and Thynell (p*K*_a_ = 5.4)^33^ suggests that HONNOH decomposition may prevail over HONNO^–^ decomposition up to significantly higher pH values than originally thought.

In any case, if the assumed HNO acid-base dimerization mechanism is correct, the kinetic isotope effect (KIE) of the respective rate-determining decomposition steps forming N_2_O should offer a straightforward way for an easy-to-reproduce ^15^N-site preference laboratory reference standard from a chemical reaction. However, to the best of our knowledge, neither the formation of N_2_O through the *cis* pathway nor the decomposition of *cis*-hyponitrite anion (or *cis*-hyponitrous acid) has been experimentally verified, nor have the molecular structures of these intermediates been thoroughly discussed in terms of KIE and equilibrium isotope effects (EIEs) and the resulting isotopomer preferences. Figure 1 illustrates the key features of the overall HNO dimerization mechanism for N_2_O formation via the decomposition of *cis*-HONNO^–^ and *cis*-HONNOH intermediates as well as their acid-base equilibria. Following the initial dimerization of HNO, several protonation and deprotonation steps (see Figure 3 in ref 31) yield *cis*-HONNO^–^. Ultrafast intramolecular hydrogen transfer, taking place on a subpicosecond time scale (see below), connects the two *cis*-HONNO^–^ isotopomers. This ultrafast equilibration overwrites possible isotopomer preferences from previous reaction steps, such that they can be neglected to predict the ^15^N-site preference in the finally generated N_2_O. The anionic *cis*-HONNO^–^ (hereinafter often referred to as A^–^) is in equilibrium with its protonated neutral form HONNOH (referred to as HA), giving rise to a pronounced pH dependence of the ^15^N-site preference in N_2_O. In contrast to previous work, the scheme in Figure 1 also includes the thermodynamically less favored open, non-hydrogen-bridged form of *cis*-HONNOH that is necessary for the interconversion of the two isotopomeric forms of the hydrogen-bridged *cis*-HONNOH. An open form of *cis*-HONNO^–^ also exists, but can be safely neglected as it is not a necessary intermediate for the equilibration of the two *cis*-HONNO^–^ isotopomers and as the chemical equilibrium, with Δ_r_ *G* ≈ 21 kJ mol^–1^ (see below), is heavily on the side of the energetically favored hydrogen-bridged form.

**Figure 1:**
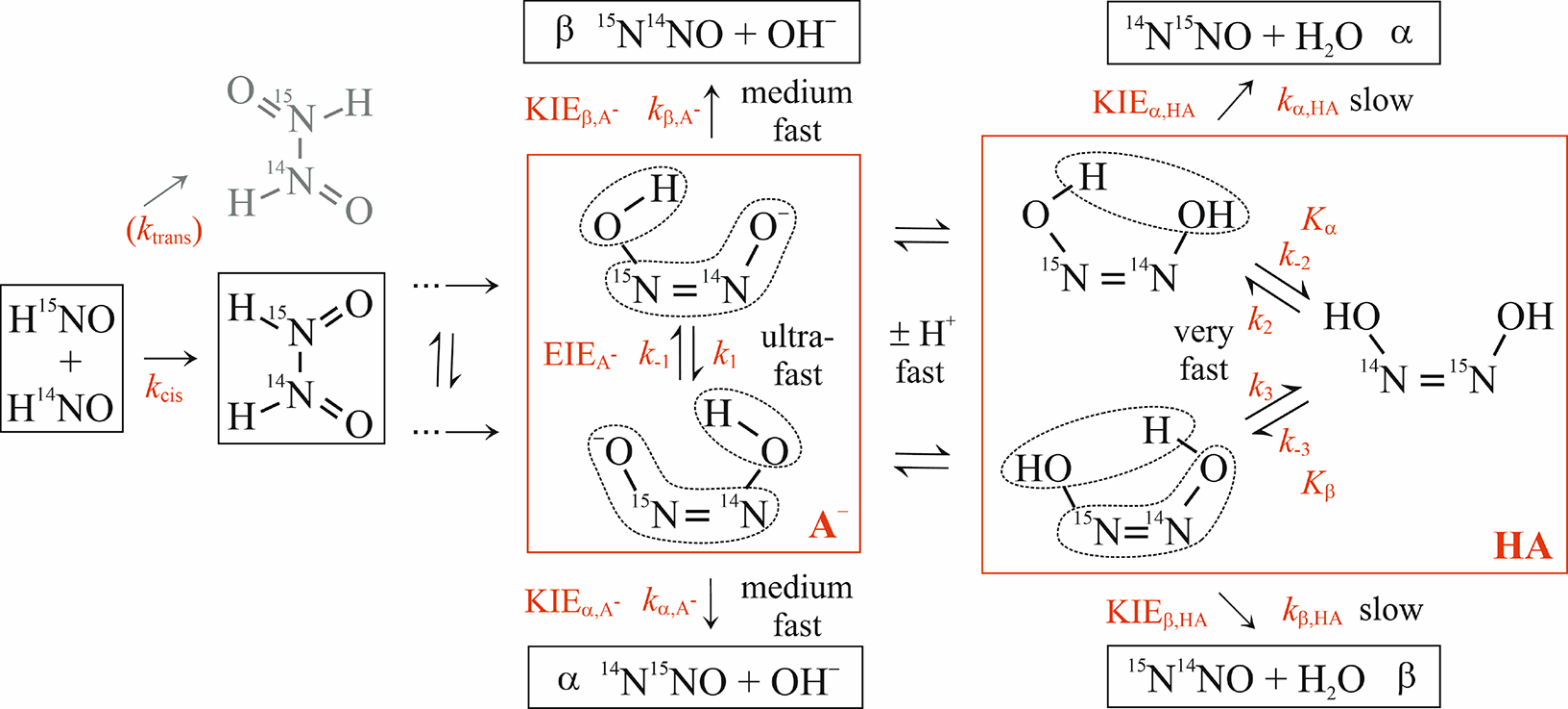
Kinetic scheme of N_2_O formation from HNO dimerization reactions through *cis* intermediates. The designation of the time scales of the different processes (slow to ultrafast) is based on rate constant calculations performed in this study. Acid-base equilibria strongly link the anion (A^–^) and neutral (HA) intermediates and determine the pH and temperature dependence of the overall site preference δ^15^N^SP^ of N_2_O formation. A detailed potential energy surface (PES) for the anion and neutral decomposition is provided in Figures 2 and 3, respectively. The different roles of the equilibrium isotope effects (EIEs), kinetic isotope effects (KIEs), equilibrium constants (*K*), and rate constants (*k*) for the overall site preference δ^15^N^SP^(N_2_O) are outlined in the Results and Discussion sections.

In view of the rather complex equilibria shown in Figure 1, it is not clear which process is most relevant for describing the overall isotopomer ratio of N_2_O from HNO dimerization. Therefore, a first objective of this study is to develop a comprehensive analytical model capable of predicting the ^15^N-site preference of N_2_O formation under synthesis conditions with varying pH and temperature. Key KIEs and EISs for N_2_O formation have been identified and described by theoretical means using ab initio calculations and transition state theory (TST). The determined molecular structures, rotational constants, and vibrational frequencies provide information about the isotopic preferences, and the rate constants reveal the time scales of the relevant equilibria and decomposition steps, hence allowing us to predict the overall δ^15^N^SP^ from first principles.

A second objective of this work is to accurately measure the absolute isotopomer ratio of N_2_O from a chemical reaction involving HNO dimerization at low pH. Such data should contribute (i) to validate the theoretically predicted preference of HNO dimerization via the *cis* intermediates,^31^ (ii) to substantiate the commonly accepted value of SP ≈ +30‰ stated by Toyoda et al.,^15^ and as such (iii) to assess HNO dimerization as a suitable laboratory reference standard for N_2_O site preference. Providing an easy-to-reproduce N_2_O source with known δ^15^N^SP^ contributes to the ongoing research efforts to improve the comparability of laboratory results on source characterization and is a major challenge still hampering the progress in N_2_O isotope research.^5,7,9,10^

Finally, a third aim of this work is to establish frequency-comb spectroscopy as an alternative to IRMS, FTIR, and QCLAS measurements for accurate δ^15^N^SP^ determination based on [^14^N^15^NO]/[^15^N^14^NO] ratio measurements. In contrast to IRMS, absorption spectroscopy—using either laser-based or FTIR methods—is inherently selective for different isotopomers. Several spectroscopic techniques using continuous wave (CW) lasers have been developed and used for N_2_O isotopomer measurements.^38–46^ However, laser spectroscopic detection schemes using narrow bandwidth tunable CW lasers, such as in QCLAS, are typically limited to the analysis of only one selected absorption line for each isotopomer, hence increasing the susceptibility to changes in analytical conditions and uncertainties in the underlying absolute absorption line strengths. In this context, recent advancements in frequency comb sources and comb-based spectroscopic techniques offer new opportunities for broadband precision molecular spectroscopy.^47–54^ The unique combination of large bandwidth, high spectral resolution, and high frequency accuracy provided by frequency combs makes them ideal for high-precision state-resolved measurements of the different isotopocules.^55–59^ In particular, the use of frequency comb spectroscopy enables straightforward state-to-state correlations of absorptions of different isotopomers by selecting multiple line pairs with the same rotational quantum numbers, thereby making the measurements of δ^15^N^SP^ values less affected by changes in temperature, gas matrix effects,^60^ and potential spectral interference by other absorbing species. Compared to conventional FTIR spectroscopy approaches, which are based on incoherent light sources with a relatively low spectral resolution of 0.011 cm^–1^,^8^ the high spectral resolution and accuracy of frequency-comb sources overcome the need for spectral corrections, in particular at preferred low pressures for isotopomer selective measurements, where the line widths are narrow.

## 2 Computational Details

### 2.1 Ab Initio Calculations

Quantum chemical calculations were performed using the Gaussian 09 suite of program.^61^ The density functional theory (DFT) method was employed using the B3LYP functional with a variety of Dunning’s correlation consistent basis sets, including aug-cc-pVDZ,^62^ aug-cc-pVTZ,^63^ and aug-cc-pVQZ,^64^ as well as the split-valence triple-ζ basis sets with different sets of polarization and diffuse functions, including 6-311++G­(d,p) and 6-311++G­(df,pd).^65^ DFT was used to provide a good balance between accuracy and computational cost, and different levels of basis sets were used to systematically improve the accuracy. Solvation effects were taken into account implicitly by applying different polarization continuum models, including CPCM and IEFPCM, using UFF cavities.^66^ After optimization, harmonic frequency analysis ensured that the minimum and transition state (TS) structures have zero and one imaginary frequency, respectively. TS structures were further checked for their correct assignment by following the intrinsic reaction coordinate to connect the respective minimum structures. Note that we report the calculated energies with a precision that far exceeds the absolute accuracy of the underlying ab initio model. Within the Born–Oppenheimer approximation, isotopic substitution does not alter the potential energy surface (PES), but by using the same ab initio electronic structure model, one can still reliably uncover the small isotope-specific relative energy changes arising from nuclear motion with the necessary numerical precision.

### 2.2 Kinetic Isotope Effects

To quantify the effect of isotopic exchange on equilibria and rate constants, TST can be utilized together with the spectroscopic Teller–Redlich product rule, where the latter links isotopic substitution to shifts in vibrational frequencies.^67^ For a chemical reaction such as A + B ⇌ AB^‡^ → P, the ratio of the rate constants of two isotopocules A and A^*^ can be expressed as

$$\frac{k_{A}}{k_{A^{*}}}=\frac{K_{A}^{‡}}{K_{A^{*}}^{‡}}=\frac{\frac{q_{A^{*}}}{q_{A}}}{\frac{q_{A^{*}B^{‡}}}{q_{{\mathrm{AB}}^{‡}}}}e^{\left(-(\Delta E_{0,A}^{‡}-\Delta E_{0,A^{*}}^{‡})/k_{B}T\right)}$$ 3

with the equilibrium constants *K*_A_^‡^ and $K_{A^{*}}^{‡}$ corresponding to the A + B ⇌ AB^‡^ and A^*^ + B $⇌A^{*}B^{‡}$ pre-equilibria, *q* to the state sums of the educts and the TS, and $\Delta E_{0,A}^{‡}-\Delta E_{0,A^{*}}^{‡}$ to the difference of the zero-point energy (ZPE)-corrected barrier heights. By applying the Teller–Redlich product rule, and using the reduced partition functions as defined by Bigeleisen-Mayeran,^68^ an expression for the KIE can be derived:

$$\mathrm{KIE}=\frac{\nu_{A}^{‡}}{\nu_{A^{*}}^{‡}}\times \prod_{i}^{3N^{‡}-7}\frac{u_{i,A}^{‡}}{u_{i,A^{*}}^{‡}}\prod_{i}^{3N-6}\frac{u_{i,A^{*}}}{u_{i,A}}\times \prod_{i}^{3N^{‡}-7}\frac{1-e^{\left(-u_{i,A^{*}}^{‡}\right)}}{1-e^{\left(-u_{i,A}^{‡}\right)}}\prod_{i}^{3N-6}\frac{1-e^{\left(-u_{i,A}\right)}}{1-e^{\left(u_{i,A^{*}}\right)}}\times \prod_{i}^{3N^{‡}-7}\frac{e^{\left(-u_{i,A^{*}}^{‡}/2\right)}}{e^{\left(-u_{i,A}^{‡}/2\right)}}\prod_{i}^{3N-6}\frac{e^{\left(-u_{i,A}/2\right)}}{e^{\left(-u_{i,A^{*}}/2\right)}}$$ 4

where ν_A_^‡^ and ν_A^*^_^‡^ are the imaginary frequencies of the TSs, corresponding to the reaction coordinate, and *u*_*i*_ = *h*ν_*i*_/*k*_B_ *T* with *h* and *k*_B_ are Planck’s and Boltzmann’s constants. The product is taken over the different vibrational frequencies ν_*i*_ of the *i*th modes of the molecule. Note that at the high temperature limit, the expression reduces to the ratio of the imaginary frequencies because all remaining terms cancel out and result in one. A similar expression holds for the EIE of an isotope exchange reaction A + B^*^ ⇌ A^*^ + B.^68^

We applied the Bigeleisen equations, such as eq 4, using the program ISOEFF98 from the Paneth group,^69^ both to quantify the intrinsic KIEs and EIEs of *cis-* and *trans*-hyponitrite as well as *cis*-hyponitrous acid. Here, the input frequencies for the vibrational modes were taken from the ab initio calculations for the all-^14^N isotopic species. Additionally, we calculated rate constants by direct application of conventional TST as implemented in the Gaussian Post Processor (GPOP) program.^70^ Both for the GPOP and ISOEFF98 approaches, a scaling factor of 0.9687 for vibrational frequencies and corresponding zero-point energies, as recommended by Merrick et al.^71^ was applied. First of all, direct TST calculations yield absolute values for the rate constants, allowing us to address the time scales of the ensuing chemical reactions. But in addition, by using the ab initio results from repeated calculations for the two ^14^N^15^N isotopomers, the ratios of the respective rate constants can also be used to determine the isotopomer preferences. Compared to ISOEFF98, which relies on the calculations of the vibrational frequencies using Hessian modifications starting from one particular isotopic species, rate constant-based calculations of the KIEs and EIEs are performed separately for the different isotopomeric species and, as such, also take into account subtle isotopic changes in the solvent effects. Fully TST-based calculations can also be beneficial to overcome some of the approximations made in deriving the Bigeleisen equation (e.g., allowing for vibrational anharmonicities, internal rotations, and higher-level quantum-mechanical tunneling corrections). However, in practice, care needs to be taken to ensure high numerical precision of all calculation steps, as the isotopic effects on the absolute rate constants are small. In the TST calculations, tunneling effects were accounted for by assuming one-dimensional asymmetric Eckart-type potentials,^72^ whereas the ISOEFF98 values were subsequently corrected by applying the common, but more approximate, Wigner tunneling correction factor:^73^

$$\Gamma_{\mathrm{tunnel}}=(1+(u_{A}^{‡}{)}^{2}/24)/(1+(u_{A^{*}}^{‡}{)}^{2}/24)$$ 5

Note that we report rate constants with more significant digits than those warranted by the absolute accuracy of the underlying TST model. This is necessary to resolve the small changes with isotopic substitution. While the absolute values mainly serve to indicate the time scales of the respective reaction steps, the isotope-induced changes in the rate constant values—arising from modifications of the molecular degrees of freedom—can be predicted with high precision.

## 3 Experimental Methods

### 3.1 Sample Preparation

Acid-catalyzed amine-borane reduction of nitrite^29^ in an acidic medium served as a source of N_2_O with a certain isotopomeric signature.

H14NO2+H15NO2→−(CH3)3N,B(OH)3,H2O(CH3)3N·BH3,H+H2+N2O14,15 6

According to Bell and Kelly,^29^ under acidic conditions, selective reduction of sodium nitrite yields HNO, which further dimerizes according to eq 2 to form N_2_O. Such a chemical reaction can provide a large volume of N_2_O in the gas phase, suitable as a transferable standard, and it is easy to reproduce. Additionally, it yields a δ^15^N^SP^ value that is independent of the isotopic composition of the substrate, as the initial dimerization step (see Figure 1) involves the formation of a symmetric OHNNHO dimer, while the KIE is governed by the decomposition of the asymmetric *cis*-HONNO^–^ and *cis*-HONNOH intermediates. Furthermore, as will turn out below, the KIE of this reaction is only weakly temperature dependent (about −0.2‰ for a temperature increase of 1 K), such that uncertainties in the synthesis temperature are less problematic.

In the sample preparation, we performed a selective reduction of isotopically labeled sodium nitrite (Na^15^NO_2_, 98%, Sigma-Aldrich; Na^14^NO_2_, 98%, Sigma-Aldrich) using stoichiometric amounts of trimethylaminoborane (97%, Sigma-Aldrich) in an acidic medium (pH ≈ 0.6) of 10% dioxane in water. The use of isotopically labeled samples allows spectral measurement at low pressure, hence circumventing the effects of strong pressure broadening that would require more sophisticated line shape models in the analysis of the spectra. A total of six syntheses were conducted at four different temperatures of 278, 296, 316, and 336 K, which were maintained by submersing the reaction vessel in a temperature-controlled water bath with an estimated uncertainty of ± 1 K. For all the syntheses, around 50:50 of ^15^N:^14^N-sodium nitrite was used to ensure a similar abundance of all the isotopocule products: ^14^N^14^NO, ^14^N^15^NO (α-isotopomer), ^15^N^14^NO (β-isotopomer), and ^15^N^15^NO. The produced gases were stripped from the reaction solution in a continuous N_2_ flow for about 1 h to ensure quantitative collection of all formed N_2_O. After passing a potassium hydroxide trap to remove CO_2_ and a dry ice-cooled trap to remove water, N_2_O was completely condensed in a liquid nitrogen-cooled trap for further spectroscopic analysis.

### 3.2 Spectroscopic Analysis

For spectroscopic analysis, spectra of rovibrational transitions of the relatively strong ν_1_ + ν_3_ vibrational band of N_2_O were measured using a Fourier transform spectrometer (FTS) based on an optical frequency comb source with ∼360 cm^–1^ bandwidth around 3450 cm^–1^.^53,59^ For details of the spectroscopic setup and spectral analysis, we refer to our recent work.^59^ Briefly, the comb^74^ was produced by difference-frequency generation in a MgO:PPLN crystal between pump and signal originating from a single Yb:fiber oscillator and was thus inherently offset-frequency-free. The repetition rate (*f*_rep_ = 125 MHz) of the comb was stabilized at the fourth harmonic to a tunable RF source referenced to a GPS-disciplined Rb oscillator.

The sample was held in a 10 cm-long single-pass cell with the temperature stabilized at 295.95 K. The cell temperature was monitored using a Pt100 platinum resistance thermometer placed in close contact with the measurement cell. The sample pressures were in the range of 2.6–6.8 mbar, chosen to yield about 50% fractional absorption for the strongest transitions. This was deemed suitable in order to obtain a good signal-to-noise ratio (SNR) while ensuring the validity of the Beer–Lambert law, which was essential for accurate retrieval of isotopomer ratios. Absorption spectra of N_2_O were measured using the subnominal resolution approach^75,76^ in the FTS to achieve comb-mode-limited resolution. The final spectra were obtained by interleaving spectra recorded with 4 different repetition rates, yielding a sampling point spacing of 31 MHz in the optical domain, where the Doppler full-width-at-half-maximum (fwhm) is ∼190 MHz or 0.0063 cm^–1^ at 296 K. Each spectrum at a given repetition rate was averaged 550 to 800 times and was normalized to a background spectrum measured with the cell evacuated. For 800 spectra at each *f*_rep_ step, the total acquisition time was 4 h. Remaining etalon artifacts in the baseline were removed by fitting a fifth-order polynomial and a sum of sine terms. The SNR for the strongest lines in the final spectra was 970:1.

## 4 Results

Dimerization of HNO in aqueous solution is assumed to proceed via a series of fast proton transfer reactions following the *cis*-pathway.^31–33^ N_2_O formation can take place from both *cis-*HONNOH and *cis-*HONNO^–^, which form the acid–base pair *cis-*HONNOH ⇌ *cis-*HONNO^–^ + H^+^. Therefore, the theoretical prediction of δ^15^N^SP^(N_2_O) must account for all reactions and equilibria shown in Figure 1. In the following sections, in a step-by-step fashion, we will first introduce the ab initio and kinetic calculations for the decomposition of the *cis*-hyponitrite and *cis*-hyponitrous acid and then bring the results together in the form of an approximate analytical model that accounts for the key isotopic effects governing the site-preference of the generated N_2_O. A similar procedure is used to analyze the alternative *trans*-pathway. Experimental measurement of absolute δ^15^N^SP^(N_2_O) values at variable temperature will be presented, compared to the analytical model predictions, and discussed with regard to the application of HNO dimerization as a laboratory reference standard.

### 4.1 *cis*-Hyponitrite Decomposition

The PES of the decomposition of the *cis*-hyponitrite anion is schematized in Figure 2. The total electronic energies (*E*_el_), including ZPE correction, and the energy values relative to the α isotopomer of *cis*-hyponitrite, Δ*E*_rel_, together with the total free energies, Δ*G*, and relative free energies, ΔΔ*G*, are listed in Table 1. The symmetric nature of the PES of the two isotopomers is a consequence of the Born–Oppenheimer separation, where isotopic exchange does not alter the electronic PES. With a TS barrier for the intramolecular hydrogen transfer of merely 6.06 kJ/mol, isomerization between reactant complexes (RC1 and RC2) of the α and β isotopomers becomes ultrafast, and with ΔΔ*G* = 24 J/mol the EIE has a direct effect on the observed N_2_O site preference as well.

**Figure 2:**
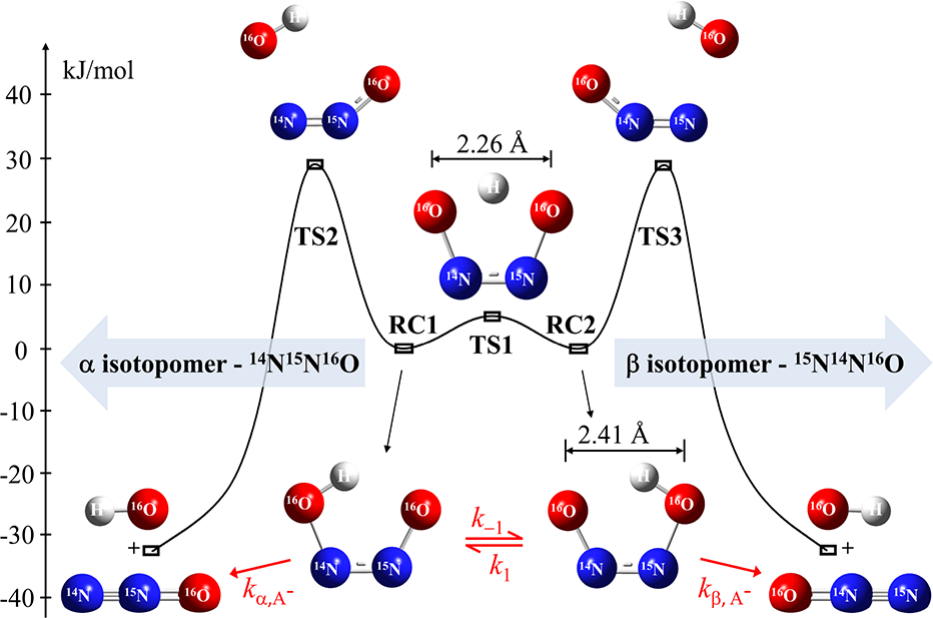
Reaction coordinate diagram for *cis*-HONNO^–^ (A^–^, see also Figure 1) decomposition forming the α and β N_2_O isotopomers. The structures of the reactant complexes (RC), transition states (TS), and products are shown. The isomerization involves intramolecular proton transfer through TS1, while the dissociation reaction involves a migration of the entire O–H moiety through TS2 and TS3 for the α and β pathways, respectively. Relative total electronic energy values, including ZPE, are based on IEFPCM-B3LYP/aug-cc-pVTZ level of theory.

**Table 1:** Total Electronic Energies, *E*_el_, Including ZPE, and Relative Energies, Δ*E*_rel_, Together with the Total Free Enthalpy, Δ*G*, and Relative Free Enthalpies, ΔΔ*G,* for Molecular Structures Calculated at the B3LYP/aug-cc-PVTZ Level of Theory^a^

*cis*-hyponitrite decomposition	*E*_el_ + 684400	Δ*E*_rel_	Δ*G* + 684400	ΔΔ*G* = Δ*G*_rel_
*cis*-HO^14^N^15^N^16^O^–^	9.127	0.000	–56.416	0.000
*cis*-HO^15^N^14^N^16^O^–^	9.158	0.031	–56.392	0.024
TS1	15.217	6.059	–49.994	6.398
TS2	43.419	34.292	–25.012	31.404
TS3	43.544	34.417	–24.910	31.506
^14^N^15^N^16^O + OH^–^	–28.826	–37.953	–125.895	–69.479
^15^N^14^N^16^O + OH^–^	–28.648	–37.775	–125.782	–69.366
*cis*-hyponitrous acid decomposition	*E*_el_ + 685500	Δ*E*_rel_	Δ*G* + 685500	ΔΔ*G* = Δ*G*_rel_
*sym*-HONNOH	–59.145	0.000	–124.472	0.000
*cis*-HO^14^N^15^N^16^OH	–56.024	3.121	–121.873	2.599
*cis*-HO^15^N^14^N^16^OH	–56.392	2.752	–121.865	2.607
TS1’	–29.174	29.971	–94.439	30.033
TS2’	–29.184	29.961	–94.449	30.023
TS3′	6.840	65.985	–58.611	65.861
TS4’	6.926	66.071	–58.546	65.926
^14^N^15^N^16^O + H_2_O	–232.678	–173.533	–332.404	–207.932
^15^N^14^N^16^O + H_2_O	–232.508	–173.363	–332.291	–207.819

a All energies are given in kJ mol^–1^. The polarizable continuum model (PCM) with the integral equation formalism variant (IEFPCM) was used to account for solvation effects.

As illustrated in Figure 2, the *cis*-HONNO^–^ structure depicts strong hydrogen bonding with a very short donor–acceptor distance of 2.41 Å. Actually, as indicated by the 0.15 Å shorter O–O distance, the symmetric TS structure enables even stronger hydrogen bonding than that of the asymmetric *cis*-HONNO^–^. Although hydrogen bonding plays a critical role in stabilizing these strained structures, another interaction involving n­(N’) → σ*­(N–O) needs to be considered as well,^31^ similar to the one discussed in the molecular *cis*-hyponitrous acid.^77^ In our case, this combined effect leads to the saddle-point geometry of TS1. Along the reaction coordinate, the hydrogen bonds in the RCs vanish, and the structure follows an energetically favorable relaxation through a dissociation step involving N–O bond cleavage and migration of the OH moiety, along with the formation of N_2_O. For the α isotopomer, dissociation takes place over transition state TS2 with an imaginary frequency of 332.47i cm^–1^ and an activation barrier of Δ*E*_rel_ = 34.292 kJ/mol, for the β isotopomer pathway via TS3 with an imaginary frequency of 328.84i cm^–1^ and an activation barrier of 34.417 kJ/mol. The energy maxima of these TSs are predominantly connected with N–O bond dissociation (a process clearly associated with a primary isotope effect) and the reorganization of the N_2_O fragment from a bent to linear structure (a process constituting a more subtle secondary isotope effect). Excluding tunneling effects, a simple TST estimate based on the Boltzmann factors using the effective ΔΔG difference of both barriers yields a KIE of about 32‰ close to room temperature (Boltzmann factors are exp­(78/*RT*) = 1.0312–1.0335 for *T* = 295 ± 10 K).

To accurately predict the intrinsic ^15^N-site preference value for N_2_O formation from the anion intermediate, δ^15^N_A^–^_^SP^, not only does the KIE of the N–O bond cleavage alone, but in particular the hydrogen transfer mediated isomerization equilibrium needs to be accounted for as well. Despite the small difference in ΔΔ*G* values of merely 24 J/mol, a significant EIE on the order of 10‰ arises close to room temperature (Boltzmann factors are exp­(24/*RT*) = 1.0095–1.0102 for *T* = 295 ± 10 K). The overall kinetic scheme that can describe the formation of the α and β isotopomers of N_2_O from the *cis*-hyponitrite intermediates is as follows (see also Figure 1):

(HO14N15NO−⇌k1k−1HO15N14NO−)N14NO15+OH−←kα,A−(···)→kβ,A−OH−+N15NO14 7

With its very low activation barrier (ΔΔ*G*) of about 6.4 kJ/mol for the intramolecular hydrogen transfer compared to the decomposition step (∼31 kJ/mol), the equilibration of HO^14^N^15^NO^–^ and HO^15^N^14^NO^–^ is expected to be fast, and hence the equilibrium will not be unbalanced by the only slightly different rates of the α and β decomposition steps. Therefore, the overall site preference for N_2_O from *cis*-hyponitrite decomposition is simply made up by the site preferences resulting from KIE and EIE (for a derivation see Supporting Information B.i) according to

δ15NA−SP=[N14NO15][N15NO14]−1=(kα,A−kβ,A−×k1k−1)−1=(KIE15N,α,A−KIE15N,β,A−×EIE)−1≈δ15NKIE,A−SP+δ15NEIE,A−SP 8

Here, ${\mathrm{KIE}}_{15_{N,\alpha ,A^{-}}}$ and ${\mathrm{KIE}}_{15_{N,\beta ,A^{-}}}$ specify the kinetic isotope effects regarding a single ^15^N-exchange of the central, α, or the terminal, β, position in ^14^N^14^NO, respectively. Nonvariational TST was applied to calculate the rate constants *k*_α, A^–^_, $k_{\beta ,A^{-}}$ , as well as *k*_1_ and *k*_–1_. Values based on the B3LYP/aug-cc-PVTZ calculations with and without tunneling correction are listed in Table 2 for *T* = 297 K and for other experimental temperatures ranging from 278 to 336 K in the Supporting Information (Tables S1 and S2). Indeed, the relaxation time of the intramolecular hydrogen transfer is ultrafast (τ_relax_ = 1/(*k*_1_ + *k*_–1_) = 402 fs), whereas the decomposition takes place medium fast with a thermal lifetime of $\frac{1}{k_{\alpha ,A^{-}}}\approx \frac{1}{k_{\beta ,A^{-}}}=53,\mathrm{ns}$ . In addition, it can be observed that the tunneling correction is significant and adds to the overall site preference. Consistent with the involved tunneling masses, tunneling contributes only 12% to the rate constant of the decomposition step but leads to a 3.1-fold increase in the rate of intramolecular hydrogen transfer. The calculated rate constants, including tunneling correction, can be directly used to determine the intrinsic ^15^N-site preference of the anionic intermediate, δ^15^N_A^–^_^SP^, according to eq 8

**Table 2:** Rate Constants, *k*, Without and With Tunneling Correction, Assuming One-Dimensional Eckart^72^ Type Potential at 297 K for the Dissociation and Isomerization Channels (IEFPCM-B3LYP/aug-cc-PVTZ Level of Theory)

rate constants:	w/o tunneling	with tunneling	isotope effects:	w/o tunneling^a^	with tunneling^a^
*cis*-hyponitrite					
$k_{\alpha ,A^{-}}$ /(10^7^s^–1^)	1.6991	1.9049	δ^15^N_KIE, A^–^_^SP^/‰	31.1/31.0	33.7/33.6
$k_{\beta ,A^{-}}$ / (10^7^s^–1^)	1.6479	1.8428			
*k*_1_/(10^12^ s^–1^)	0.4052	1.2392	δ^15^N_EIE, A^–^_^SP^/‰	9.3/9.6	9.3/9.6
*k*_–1_/(10^12^ s^–1^)	0.4089	1.2507			
*cis*-hyponitrous acid					
$k_{\alpha ,\mathrm{HA}}$ /(10^2^s^–1^)	0.7713	6.2271	δ^15^N_KIE, HA_^SP^/‰	22.3/22.7	30.0/30.4
$k_{\beta ,\mathrm{HA}}$ /(10^2^s^–1^)	0.7545	6.0456			
*k*_2_/(10^7^ s^–1^)	6.0876	7.6918	δ^15^N_EIE, HA_^SP^/‰^b^	3.3/3.1	3.3/3.1
*k*_–2_/(10^8^ s^–1^)	1.7715	2.2383			
*k*_3_/(10^8^ s^–1^)	1.7841	2.2547			
*k*_–3_/(10^7^ s^–1^)	6.1107	7.7229			

a GPOP (from rate constants)/ISOEFF98 (from Bigeleisen equation).

b ${\mathrm{EIE}}_{\mathrm{HA}}=\left(\frac{k_{2}k_{3}}{k_{-2}k_{-3}}-1\right)\times 1000‰$ .

### 4.2 *cis*-hyponitrous Acid Decomposition

The PES for the decomposition of *cis*-hyponitrous acid is illustrated in Figure 3. The total electronic energies (*E*_el_), including ZPE correction, and the energy values relative to the symmetric, open conformer (*sym*-HONNOH), Δ*E*_rel_, as well as the total free energies, Δ*G,* and relative free energies, ΔΔ*G*, are listed in Table 1. Unlike the hyponitrite anion, the PES of the neutral species features a double-well potential with a central double barrier. The symmetric *cis*-HONNOH conformer (RC1’) has the two OH groups pointing outward, lying in the O–N–N–O molecular plane. Interestingly, this symmetric conformer is more stable than the α and β conformers (RC2’ and RC3′), in which one of the OH groups is pointing inward, forming an OH···O intramolecular hydrogen bond. This surprising destabilization of the hydrogen-bridged species can be attributed to the counteracting interaction of lone pairs of electrons in the oxygen and nitrogen atoms, such as n­(N)→ σ*­(N–O) and n­(O) → π*­(N–N).^77^ As a result, a more open structure with an O–O distance of 2.45 Å is obtained for the hydrogen-bridged conformers compared to 2.40 Å in the symmetric conformer.

**Figure 3:**
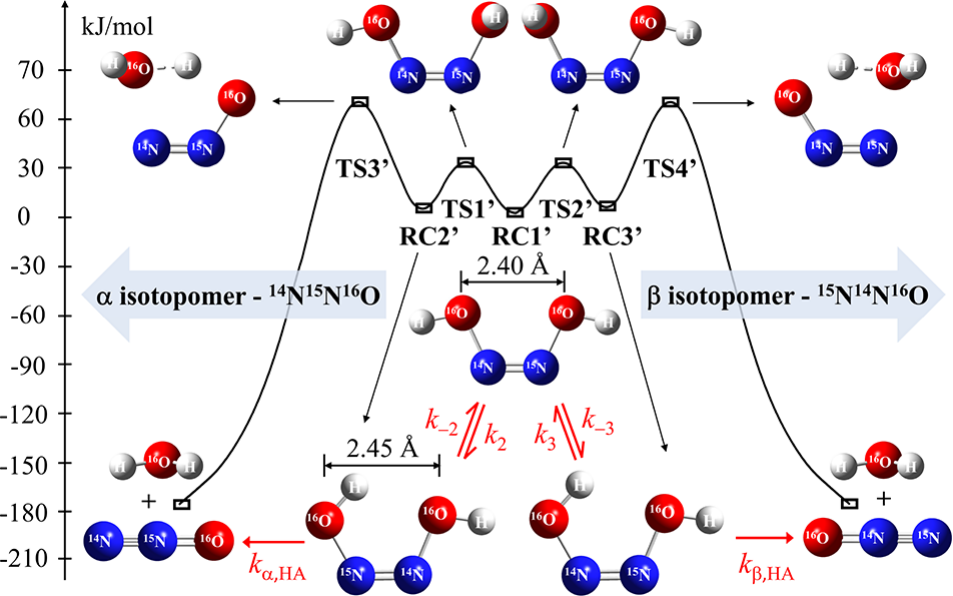
Reaction coordinate diagram for *cis*-HONNOH (HA, see also Figure 1) decomposition forming the α- and β-N_2_O isotopomers. The structures of the reactant complexes (RC’), transition states (TS’), and products are shown. The relative energy values, including ZPE, are based on calculations at the IEFPCM-B3LYP/aug-cc-pVTZ level of theory.

The rotation of the OH groups involves TSs with the H atom of the rotating OH group pointing perpendicular to the O–N–N–O plane. As can be seen from the rate constants listed in Table 2 (τ_relax_ = 1/(*k*_2_ + *k*_–2_) ≈ 1/(*k*_3_ + *k*_–3_) = 3.3 ns), the very fast isomerization takes place on the time scale of a few nanoseconds. The transition states (TS1’ and TS2’) are characterized by imaginary frequencies of 467.08i and 467.30i cm^–1^, with relative energies of 29.971 and 29.961 kJ mol^–1^ for the α- and β- pathways, respectively. The subsequent decomposition step proceeds through the elimination of a water molecule via a high-lying TS at 65.985 kJ mol^–1^ with an imaginary frequency of 1258.94i cm^–1^ for the α-pathway, and at 66.071 kJ mol^–1^ with an imaginary frequency of 1257.06i cm^–1^ for the β-pathway. Note that with a room temperature tunneling-corrected rate constant of about 6.1 × 10^2^ s^–1^ (corresponding to a lifetime of 1.6 ms), *cis*-hyponitrous acid is much more stable than the *cis*-hyponitrite anion that decomposes with a rate constant of about 1.9 × 10^7^ s^–1^ (lifetime of 53 ns). As a consequence of the 3.1 × 10^4^ slower decomposition, *cis*-hyponitrous acid decomposition can only compete with *cis*-hyponitrite decomposition at low pH values, at least four units below the p*K*_a_ of the *cis*-HONNOH/*cis*-HONNO^–^ acid–base pair.

Similar to the *cis*-HONNO^–^ intermediate, an intrinsic site preference value of the neutral intermediate, δ^15^N_HA_^SP^, can be determined based on the following equilibrium system (see also Figure 1):

(HO14N15NOH⇌k−2k2sym‐HONNOH⇌k−3k3HO15N14NOH)N14NO15+H2O←kα,HA(···)→kβ,HAH2O+N15NO14 9

Again, the equilibration of the α- and β-intermediates is fast, and the overall site preference for N_2_O from *cis*-hyponitrous acid decomposition is directly related to the site preferences resulting from KIE and EIE (for a derivation, see Supporting Information B.ii):

$$\delta^{15}N_{\mathrm{HA}}^{\mathrm{SP}}=\left(\frac{k_{\alpha ,\mathrm{HA}}}{k_{\beta ,\mathrm{HA}}}\times \frac{k_{2}k_{3}}{k_{-2}k_{-3}}\right)-1=\left(\frac{{{\mathrm{KIE}}_{15}}_{N,\alpha ,\mathrm{HA}}}{{{\mathrm{KIE}}_{15}}_{N,\beta ,\mathrm{HA}}}\times {\mathrm{EIE}}_{\mathrm{HA}}\right)-1\approx \delta^{15}N_{\mathrm{KIE},\mathrm{HA}}^{\mathrm{SP}}+\delta^{15}N_{\mathrm{EIE},\mathrm{HA}}^{\mathrm{SP}}$$ 10

Note that the equilibrium concentration ratio and with it the EIE of the equilibrium HO^14^N^15^NOH and HO^15^N^14^NOH is given by *K*_α_ × *K*_β_ = (*k*_2_ *k*_3_)/(*k*_–2_ *k*_–3_) (see also Figure 1). Rate constant values with and without tunneling correction are listed in Table 2 for *T* = 297 K and for the experimental synthesis temperatures ranging from 278 to 336 K in the Supporting Information (see Tables S3 and S4). Again, tunneling turns out to be significant. Most striking is the rate increase by a factor of 8 observed for the decomposition step, which shows that the decomposition is dominated by tunneling events, and thus, the predicted isotopomer preference is expected to sensitively depend on the assumed tunneling model.

### 4.3 Intrinsic ^15^N-Site Preferences

The rate constants from Tables 2 and S1–S4 in the Supporting Information can directly be used to calculate the KIE and EIE for N_2_O isotopomer site preference using eqs 8 and 10. Alternatively, the Bigeleisen equations and Hessian modifications as implemented in the ISOEFF98 program make it possible to estimate the isotopic effects solely on the basis of the calculated vibrational frequencies of the all-^14^N intermediates and TSs. Table 2 compares site preference values obtained from either the calculated rate constants using GPOP or the Bigeleisen equation implemented in ISOEFF98. Both methods yield consistent results within a few tenths of ‰, where the remaining small deviations are due to minor methodological differences and numerical inaccuracies. The latter are expected to be more pronounced for the rate constant-based estimates, which ultimately require a precision of the calculated absolute values of the rate constants of about 5 digits. In the following, we therefore rely mainly on the ISOEFF98 site preference values, which we, however, corrected with the GPOP-predicted Eckart tunneling effect.

Table 3 presents the corresponding temperature-dependent values, indicating that both KIE and EIE values show the expected decrease with increasing temperature. Overall, with −0.19‰/K for *cis*-HONNOH and −0.16‰/K for *cis*-HONNO^–^, the dependence is rather weak such that minor uncertainties in the synthesis temperature of the N_2_O samples are less critical for the experimental outcome. For both intermediates, the KIE dominates the overall site preference, resulting in $26.9‰<\delta^{15}N_{\mathrm{HA}}^{\mathrm{SP}}<37.9‰$ for *cis*-hyponitrous acid and $37.6‰<\delta^{15}N_{A^{-}}^{\mathrm{SP}}<47.1‰$ for the *cis*-hyponitrite anion over the considered temperature range. Overall, with predicted differences $\delta^{15}N_{A^{-}}^{\mathrm{SP}}-\delta^{15}N_{\mathrm{HA}}^{\mathrm{SP}}$ in the range of 9.2–10.7‰, N_2_O formation from *cis*-hyponitrite anion or *cis*-hyponitrous acid decomposition should be clearly distinguishable by the accurate isotopomer ratio measurements performed in this work.

**Table 3:** Temperature Dependence of the Calculated Intrinsic Isotope Effects (IEFPCM-B3LYP/aug-cc-pVTZ, ISOEFF, Including Eckart Tunneling)^a^

*T*/K	δ^15^N_KIE, HA_^SP^/‰	δ^15^N_EIE, HA_^SP^/‰	δ^15^N_HA_^SP^/‰	δ^15^N_KIE, A^–^_^SP^/‰	δ^15^N_EIE, A^–^_^SP^/‰	δ^15^N_A^–^_^SP^/‰
278	34.4	3.4	37.9	36.2	10.5	47.1
297	30.4	3.1	33.6	33.6	9.6	43.5
316	27.1	2.8	30.0	31.3	8.8	40.4
336	24.2	2.6	26.9	29.3	8.1	37.6

a The total SP values are calculated using the exact form of eqs 8 and 10.

We also analyzed the effect of the chosen basis set on the predicted site preference. Results are shown in Table S9 in the Supporting Information for selected δ^15^N^SP^ values with increasing basis set size, both for Dunning’s correlation consistent and split-valence triple-ζ basis sets. Overall, the different basis sets yield very comparable values, and no systematic trend is recognizable. With standard deviations of $\sigma \left(\delta^{15}N_{\mathrm{HA}}^{\mathrm{SP}}\right)=\pm 0.22‰$ and σ­(δ^15^N_A^–^_^SP^) = ±0.47‰, the total site preference can be estimated to be precise within a few tenths of per mille. For the sake of completeness, Tables S10 and S11 present electronic energies and imaginary frequencies for selected TSs and reactant complexes. It is evident that, unlike the almost negligible influence of the basis set on KIE and EIE, the computed energies and imaginary frequencies exhibit a notable dependency on the selected basis set. This underscores the significant role of error cancellation when evaluating both rate constant ratios and energy differences for the calculation of KIEs and EIEs.

### 4.4 *trans* HNO Dimerization

For comparison, we also calculated the expected site preference for the alternative *trans* HNO dimerization pathway. Here, as outlined by Fehling and Friedrichs,^31^ N_2_O can be generated by decomposition of the *trans*-HONNO^–^ anion only. For the protonated form *trans*-HONNOH, a simple splitting off of a water molecule is not possible; therefore, it can be considered a stable species. In a simplified manner, the site preference results from the following acid-base equilibrium (see also Figure S2 in the Supporting Information):

[trans‐HONNOH](trans‐HO14N15NO−⇌+H+[···]⇌−H+trans‐O−14N15NOH)N14NO15+OH−←kα,A−trans(···)→kβ,A−transOH−+N15NO14 11

Different from the ultrafast equilibration of the two isotopomeric species of *cis*-HONNO^–^ by H atom transfer, the isotopomer equilibrium between *trans*-HO^14^N^15^NO^–^ and *trans*-HO^14^N^15^NO^–^ is mediated by fast acid-base protonation/deprotonation steps. This results in an EIE for the *trans*-HO^14^N^15^NO^–^ ⇌ *trans*-^–^O^14^N^15^NOH equilibrium. Note that the reaction scheme in eq 11 is actually a bit too simple in this form, because several conformers of both *trans*-HONNO^–^ and *trans*-HONNOH should be accounted for as well. The *trans*-HONNO^–^ has two conformers, while *trans*-HONNOH has three conformers with respect to the orientation of the OH group, with either the OH group pointing outward or inward. For *trans*-HONNOH, the conformer with the lowest energy is the one with two OH pointing outward, with one OH rotation requiring ∼3.5 kJ mol^–1^. Just the other way round, the *trans*-HONNO^–^ conformer with the OH pointing inward is slightly more stable by 1.3 kJ mol^–1^. For a more detailed discussion of the equilibrium system and its treatment for calculating the site preference, please refer to sections *B.v* and D in the Supporting Information. Similar to the *cis* case, however, the OH rotation is taking place on a much faster time scale (∼10^–8^ s) compared to the diffusion-controlled acid-base equilibration of the different conformers (∼10^–3^ s), which in turn is still much faster than the eventual dissociation of the *trans*-HONNO^–^ anion (∼0.1 to 1 s).

As a result, the four isotopic species of *trans*-HONNO^–^ (two conformers for α and β each) can be safely assumed to be in equilibrium with each other. Accordingly, as further outlined in the Supporting Information, the overall site preference stems from a mole fraction (EIE) and rate constant (KIE) weighted contribution of the decomposition of the four conformers. Calculations on the B3LYP-aug-cc-PVTZ level of theory reveal a site preference for N_2_O formation from *trans* decomposition of $\delta^{15}N_{\mathrm{total},trans}^{\mathrm{SP}}=-7.3‰$ at *T* = 297 K with Eckart tunneling ( −5.9‰ with Wigner and −1.2‰ without tunneling correction). The contribution of KIEs and EIEs at *T* = 297 K is $\delta^{15}N_{\mathrm{KIE},trans}^{\mathrm{SP}}\approx -17‰$ and δ^15^N_EIE, *trans*_^SP^ = +10‰, and δ^15^N_total, *trans*_^SP^ slightly increases from −7.8‰ to −6.7‰ when increasing the temperature from 278 to 336 K. Clearly, with about 40−50‰ lower site preference values for the *trans*-pathway compared to the *cis*-pathway, experimental measurement of the overall site preference from HNO dimerization as performed in this work, should allow one to confirm or reject the previously assumed dominance of the *cis*-pathway.

### 4.5 pH Dependence of Total ^15^N-Site Preference

In order to derive a suitably weighted total site preference $\delta^{15}N_{\mathrm{total}}^{\mathrm{SP}}$ as a result of a simultaneous decomposition of the *cis*-HONNO^–^ and *cis*-HONNOH, next to accounting for the interconversion of the α and β isotopomers outlined in Sections 4.1 and 4.2, the acid-base equilibrium between the neutral and the anionic species needs to be considered. As the flow of reacting HNO molecules feeds only into the anionic species (Figure 1), the question arises how fast the equilibration between *cis*-HONNO^–^ and *cis*-HONNOH takes place. Two limiting scenarios are conceivable. On the one hand, for an equilibration much slower than the decomposition of the anionic species, the total site preference would be equal to the site preference $\delta^{15}N_{A^{-}}^{\mathrm{SP}}$ . On the other hand, for a correspondingly fast equilibration, the equilibrium acid-base ratio is given by the Henderson–Hasselbalch equation, pH = p*K*_a_ + log­([HONNO^–^]/[HONNOH]). As will become clear below, the second case is valid under the experimental conditions of this work.

Unfortunately, to the best of our knowledge, no experimental p*K*_a_ value has been reported in the literature for *cis*-hyponitrous acid. Fehling and Friedrichs^31^ predicted a value of 3.1 based on a proton exchange ansatz. Their calculations were based on *trans*-hyponitrous acid as a reference acid, which has a reported p*K*_a_ value of 7.18.^78^ They set this p*K*_a_ equal to the p*K*_a_ of the thermodynamically most stable conformer with the two OH groups pointing outward. However, at room temperature, the two other conformers contribute to the overall acid-base equilibria, as well. Based on B3LYP-aug-cc-PVTZ free enthalpy differences of all involved conformers, we have estimated that accounting for the different conformers would have resulted in a somewhat higher value of p*K*_a_ ≈ 3.6 for *cis*-hyponitrous acid. Yet another estimate using HONO as an alternative reference acid yielded p*K*_a_ ≈ 5.0, showing that the reference method reacts very sensitively to the selection of the respective reference acid. Actually, the latter value is largely consistent with the calculations by Zhang and Thynell,^33^ who employed a more advanced cluster-continuum model. They predicted a p*K*_a_ value of 6.14 for bare *cis*-hyponitrous acid, but the value dropped to 5.4 when four hydrogen-bonded water molecules were explicitly included in the calculation.

In the following, we rely on p*K*_a_ ≈ 5.4 from Zhang and Thynell,^33^ but allow for larger uncertainties of this key quantity. Note that using this value instead of p*K*_a_ ≈ 3.1 from Fehling and Friedrichs^31^ significantly shifts the equilibrium HONNOH ⇌ HONNO^–^ + H^+^ to the side of the neutral hyponitrous acid, such that even at the low pH of our experiments, the decomposition of the neutral hyponitrous acid can compete with the decomposition of the hyponitrite anion. Therefore, at a given pH, the overall isotopomer ratio needs to be expressed as a weighted sum of the intrinsic isotopomer ratios of the *cis*-hyponitrite anion (according to eq 8) and *cis*-hyponitrous acid (according to eq 10), where the weight accounts for both the fraction of the neutral and anion species in acid-base equilibrium as well as their respective decomposition rate constants for N_2_O formation. At low pH, the relaxation time τ of the acid-base equilibrium is short compared to the lifetimes of HONNOH and HONNO^–^. Here, the relaxation rate of the acid-base equilibrium H^+^ + A^–^ ⇌ HA is given by τ^–1^ = *k*_recombination_[H^+^] + *k*_dissociation_ ≈ *k*_diffusion_ (10^–pH^ + 10^–p*K*_a_^) for a diffusion-limited recombination step. At pH = 1, p*K*_a_ = 5.4, and with *k*_diffusion_ = 4.5 × 10^10^ M^–1^/s, set equal to the experimental value for H^+^ + acetate recombination,^79^ the relaxation time becomes 0.22 ns, which is indeed much shorter than the lifetime of HONNOH (1.6 ms) and HONNO^–^ (53 ns). Consequently, at not too high pH, fast equilibration of all involved species can be assumed and the overall δ^15^N_total_^SP^ is given by the following equation (for a full derivation see Supporting Information B.iii),

$$\delta^{15}N_{\mathrm{total}}^{\mathrm{SP}}=\frac{1}{k_{A^{-}}+k_{\mathrm{HA}}\cdot 10^{-\left(\mathrm{pH}-pK_{a}\right)}}\times \left(\delta^{15}N_{A^{-}}^{\mathrm{SP}}\cdot k_{A^{-}}+\delta^{15}N_{\mathrm{HA}}^{\mathrm{SP}}\cdot k_{\mathrm{HA}}\cdot 10^{-\left(\mathrm{pH}-pK_{a}\right)}\right)$$ 12

where *k*_A^–^_ and *k*_HA_ are the rate constants of the dissociation of the anion and the neutral species, and δ^15^N_A^–^_^SP^ and δ^15^N_HA_^SP^ are the corresponding intrinsic ^15^N-site preference values.

Note that the 10^–(pH–p*K*_a_)^ terms account for the pH-dependent equilibration between the anion and neutral species based on the Henderson–Hasselbalch equation. Using *k*_ratio_ = *k*_A^–^_/*k*_HA_, eq 12 can be rewritten as

$$\delta^{15}N_{\mathrm{total}}^{\mathrm{SP}}=\frac{1}{k_{\mathrm{ratio}}+10^{-\left(\mathrm{pH}-pK_{a}\right)}}\times \left(\delta^{15}N_{A^{-}}^{\mathrm{SP}}\cdot k_{\mathrm{ratio}}+\delta^{15}N_{\mathrm{HA}}^{\mathrm{SP}}\cdot 10^{-\left(\mathrm{pH}-pK_{a}\right)}\right)$$ 13

### 4.6 Broadband Frequency Comb Spectroscopy

The predicted total ^15^N-site preference value from eq 12 or 13 can be compared with independently determined values from high-precision frequency comb measurements in order to verify whether the mechanism of HNO dimerization follows a *cis*-pathway and which *cis* intermediate species is predominant for the observed isotope effect under the applied synthesis conditions.

Figure 4 shows the broadband high-resolution spectrum of the ν_1_ + ν_3_ band of the chemically synthesized, isotopomerically labeled N_2_O sample measured at 3.2 mbar using the optical frequency comb FTS. As shown in this Figure, the simultaneously measured absorption bands of the four generated isotopomers: ^14^N^14^N^16^O, ^14^N^15^N^16^O, ^15^N^14^N^16^O, and ^15^N^15^N^16^O have similar absorption levels, which is advantageous for an accurate determination of δ^15^N^SP^ values.

**Figure 4:**
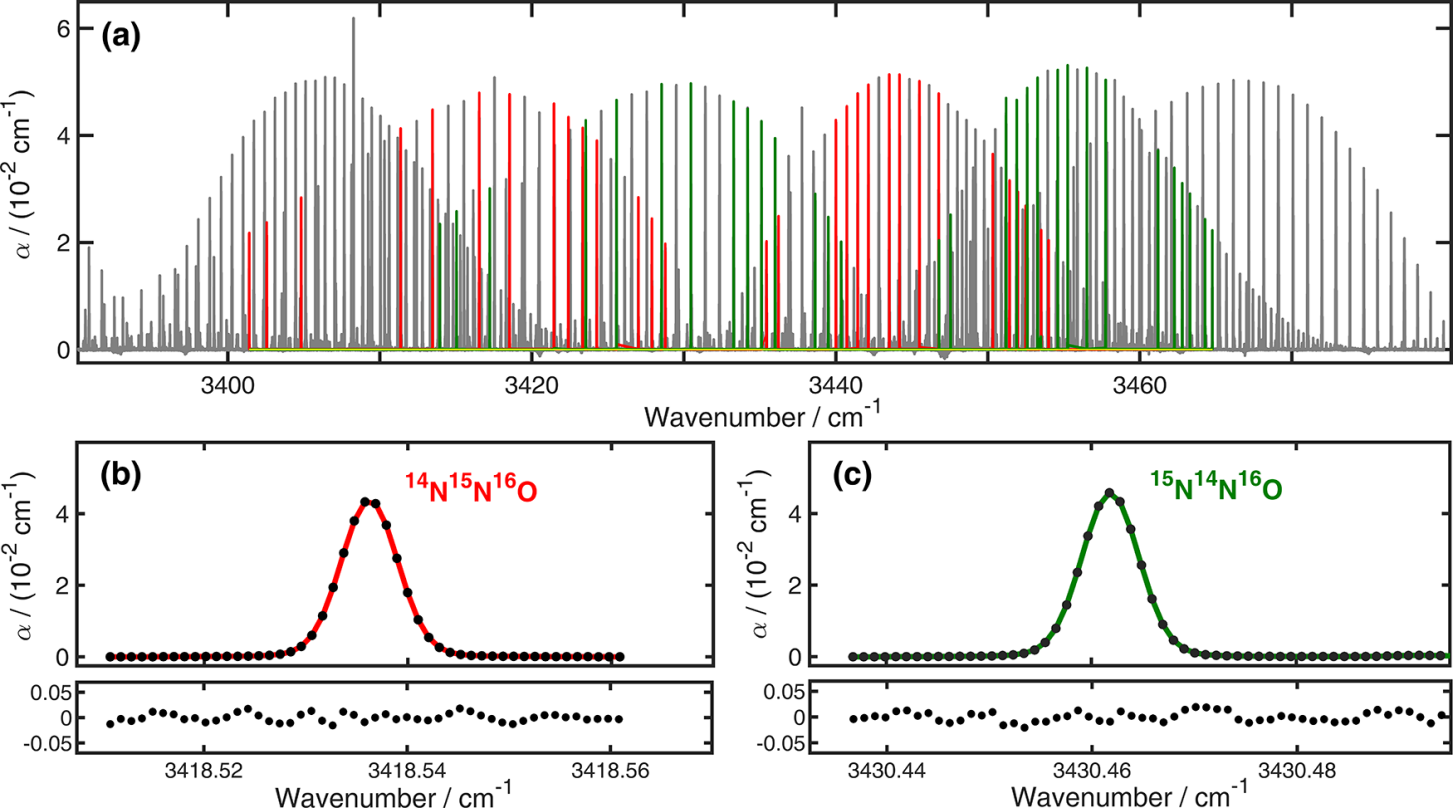
(a) Broadband high-resolution absorption coefficient, α of chemically synthesized N_2_O at 278 ± 1 K, measured using the comb-based FTS (gray) at 3.2 mbar. The ratio of ^15^N:^14^N-nitrite was 49:51. The selected line pairs for δ^15^N^SP^ determination are highlighted in red (^14^N^15^N^16^O) and green (^15^N^14^N^16^O). (b, c) Measured absorption profiles (filled circles) of a pair of P(15) transitions for the α and β isotopomers from the same synthesis at *T* = 278 ± 1 K, together with a fit of a Voigt model (red and green curves) and the respective fit residuals in the lower panels.

For the determination of δ^15^N^SP^ from the measured spectra, we first selected 30 pairs of lines for α and β isotopomers with a HITRAN2020 ^80^ line strength larger than 10^–23^ cm^–1^/(molecules cm^–2^) and sharing the same lower state rotational quantum number, *J*, between 3 and 32. We selected only spectrally well-isolated lines that were separated by at least 700 MHz from the nearest neighboring line. Some line pairs fulfilling this criterion were excluded because they occurred in regions with residual baseline problems caused by water absorption from the ambient air. The selected transitions for the α and β isotopomers are listed in the Supporting Information (Table S12), together with the difference in their lower state energies, and are highlighted in Figure 4 in red and green, respectively.

Figure 4b,c shows a pair of P(15) lines for the α and β isotopomers from the sample synthesized at 278 ± 1 K, respectively, together with the fitted Voigt line shape functions and residuals. These two lines have differences in transition wavenumber of 12 cm^–1^ and of lower state term value of only 3.39 cm^–1^. It can therefore be assumed that changes in the measurement conditions, such as the temperature, have very little effect on the determined isotopomer ratio. In this way, we analyzed 60 individual absorption profiles with line positions, Lorentzian widths, and intensities as free parameters, and Doppler full width at half-maximum fixed to 190 MHz, a value calculated at 296 K.

Table 4 summarizes the experimental δ^15^N^SP^ values determined using line-by-line fitting for the 6 syntheses at four different temperatures. The reported fit uncertainties in δ^15^N^SP^ were estimated from the square root of the diagonal elements of the covariance matrix for each Voigt profile in the line-by-line fit and were propagated to the weighted mean of the δ^15^N^SP^ values. The measurements at different synthesis temperatures serve as a critical test for both the capability of our spectroscopic technique to measure small changes in δ^15^N^SP^ and to validate the theoretically predicted temperature dependence. As shown in Table 4, the sub-‰ agreement between the δ^15^N^SP^ values determined from syntheses at the same temperature, i.e., syntheses I and II at 278 ± 1 K, and syntheses IV and V at a temperature of 316 K, is excellent.

**Table 4:** Experimentally Determined δ^15^N^SP^ Values (2σ Fit Uncertainties) for Nitrous Oxide Formation from HNO Dimerization at Different Temperatures of Synthesis at Nearly Constant pH and ^15^N Isotopic Fraction^15^R^s^ in the Substrate

synthesis	*T*/K	pH	^15^R^s^	δ^15^N^SP^/‰
I	278 ± 1	0.68	0.49	36.6 ± 1.2
II	278 ± 1	0.68	0.50	36.7 ± 1.4
III	297 ± 1	0.60	0.49	30.1 ± 1.8
IV	316 ± 1	0.60	0.50	30.0 ± 1.2
V	316 ± 1	0.56	0.50	29.1 ± 1.4
VI	336 ± 1	0.58	0.50	23.4 ± 1.2

We note that the accuracy of the experimentally derived δ^15^N^SP^ values is limited by the accuracy of the available line strengths in the HITRAN2020 database.^80^ However, we have shown recently^59^ that the relative intensities of the α and β isotopomers are consistent with this database. Moreover, as our δ^15^N^SP^ values were derived from ratios of integrated absorption coefficients of isotopomeric line pairs with matched lower-state rotational quantum numbers, this approach inherently cancels many systematic factors, including temperature, global intensity scaling, and partition function errors. Referring to our previous work,^59^ the relative intensities over the full ν_1_ + ν_3_ combination band agreed with HITRAN data on a 1.6–3.0% standard deviation level. From this full data set, we have selected 30 high-quality line pairs as described above (Table S12 in the Supporting Information). As deduced from the weighted average, the 1σ fit uncertainty for the line pair ratios turned out to be 0.37%, resulting in 0.68‰ uncertainty in the corresponding δ^15^N^SP^ value. A plot highlighting all fitted relative line pair intensity ratios with respect to HITRAN2020 line strength data is provided in Figure S3 in the Supporting Information. The reproducibility of the *single* line pair ratio measurements across all six syntheses (0.37%) is fully consistent with the average fit uncertainty, confirming an essentially statistical scatter from the line fitting procedure. However, the somewhat larger ± 0.76% weighted standard deviation of all data points (which would result in 1.39‰ in δ^15^N^SP^) reveals an additional source of uncertainty, which we attribute to residual inaccuracies of the reported HITRAN line strength data. The resulting over- and underestimation of several line pair ratios is clearly visible in Figure S3. Although such line pair-specific uncertainties can be expected to largely cancel out in the average of the 30 line pairs, a residual systematic bias in the relative scaling of ^14^N^15^NO vs ^15^N^14^NO line strengths in the HITRAN2020 database, if it exists, would propagate as an offset in δ^15^N^SP^. Such a bias, which would not show up in the scatter of the experimental data, may result from inaccuracies in the empirically refined PES and dipole moment surface (DMS) as well as the inclusion/omission of nonadiabatic and diagonal Born–Oppenheimer correction terms.^81,82^ Therefore, for a conservative error estimate including such uncertainties, we allow for a 1.5‰ error estimate for δ^15^N^SP^, in addition to the (1.2–1.8‰) 2σ fit uncertainties included in Table 4. Note that continued advances in line intensity modeling will allow this error margin to be reduced further. Progress in theoretical prediction of line intensities is breathtaking.^82^ For example, predicted absolute line intensities for CO_2_ are already approaching 0.1% accuracy,^83^ with expected isotopologue consistency on a 0.2‰ level.^84^

## 5 Discussion

Figures 5 and 6 summarize the results and compare the outcome of this study to the few available site preference data from the literature.

**Figure 5:**
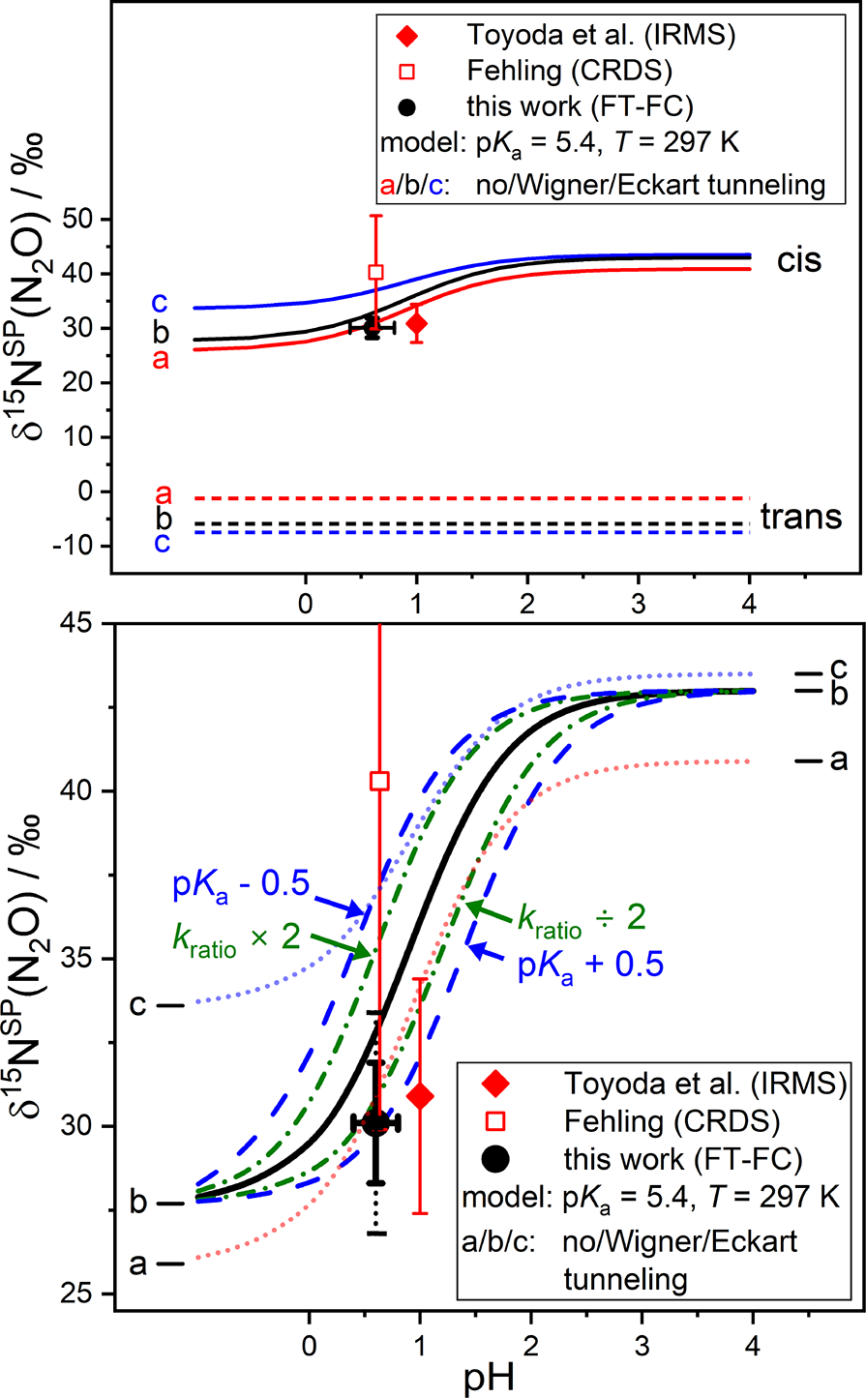
Comparison of experimental and theoretically predicted ^15^N-site preference of N_2_O at different pH values of the synthesis solution for HNO dimerization. Symbols represent the experimental data of this work (black dot, with the inner error bar corresponding to 2σ fit uncertainty and the outer bar allowing for an estimated ±1.5‰ uncertainty for potential systematic relative isotopomer inconsistency in HITRAN2020), IRMS measurements (red diamond),^15^ and NIR-CRDS (red square).^85^ Upper plot: Theoretically predicted pH-dependent curves for fixed p*K*_a_ = 5.4 and temperature *T* = 297 K for N_2_O formation from the *cis* pathway and *trans* pathway (label *a*: without, *b*: with Wigner, and *c*: with Eckart tunneling correction). Lower plot: Enlargement of the results for the *cis* pathway includes an evaluation of scenario *b,* focusing on how sensitive the predicted site preference is to variations in p*K*_a_ and *k*_ratio_. The blue-dashed curves indicate the occurring shift of the pH-dependent site preference when assuming Δ­(p*K*_a_) = ± 0.5. It is a result of the equilibrium between *cis*-hyponitrous acid (dominating at low pH) and *cis*-hyponitrite (dominating at high pH). The green dash-dotted curves reveal a similar behavior when changing the rate constant ratio of *cis*-hyponitrous acid and *cis*-hyponitrite decomposition forming N_2_O.

**Figure 6:**
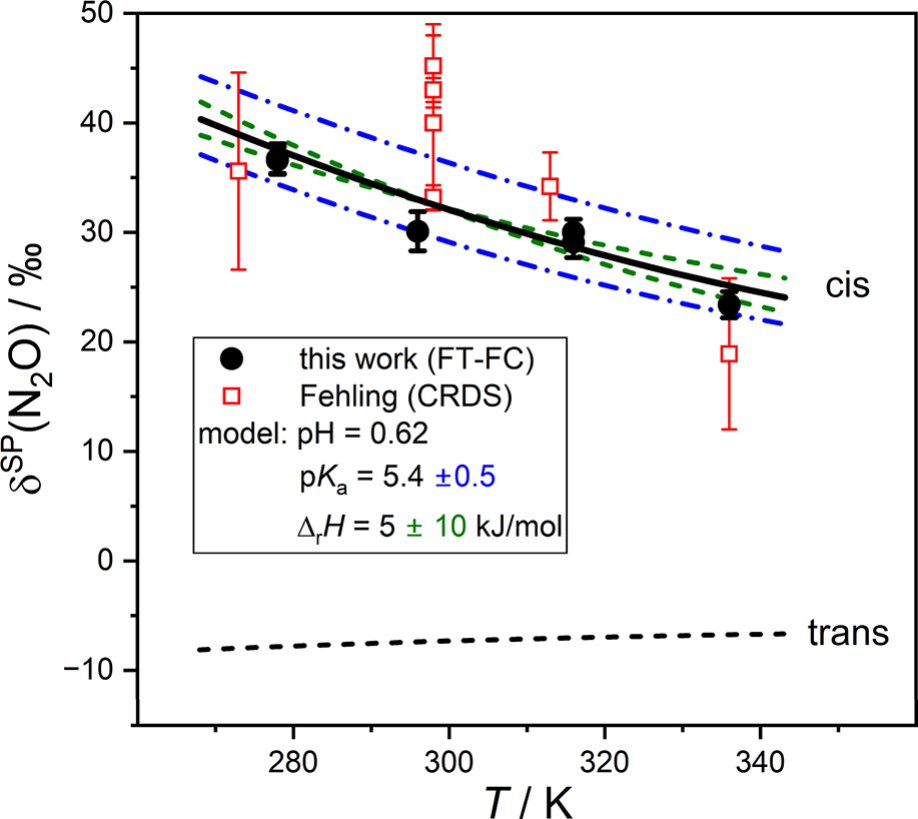
Temperature dependence of δ^15^N^SP^(N_2_O). Experimental values obtained from mid-IR frequency comb spectroscopy (this work, black dots) and near-IR CRDS (open squares)^85^ as a function of synthesis temperature, together with theoretically derived values using the model from eq 13. The solid black curves assumes p*K*_a_ = 5.4, pH = 0.62, δ^15^ *N*_HA, *cis*_^SP^ = 27.7‰, δ^15^ *N*_A^–^, *cis*_^SP^ = 43.0‰, and δ^15^ *N*_A^–^, *trans*_^SP^ = – 7.4‰ at *T* = 297 K. The temperature dependence of the δ^15^ *N* ^*SP*^ values has been adopted from Eckart tunneling-corrected values, and the temperature dependence of p*K*_a_ is calculated from van’t Hoff’s equation, assuming a reaction enthalpy of Δ_r_ *H* = 5 kJ/mol. The blue dashed curves represent the ± 0.5 p*K*_a_ boundaries, while the green dashed curves represent curves with the enthalpy of reaction varied by ± 10 kJ/mol.

### 5.1 *cis* versus *trans* HNO Dimerization

In Figure 5, the theoretically predicted and experimental site preference values are shown as a function of the pH value set for N_2_O synthesis from HNO dimerization at room temperature (*T* = 297 K experiment). In the upper panel of Figure 5, both the predicted δ^15^N^SP^ from HNO dimerization taking place via the *cis* and *trans* pathways are outlined as solid and dashed curves, respectively. The red, black, and blue curves correspond to calculations without (red, label a) and with either Wigner (black, label b) or Eckart tunneling (blue, label c) correction. Clearly, our measurement (black filled circle, δ^15^N^SP^ = 30.1‰) is consistent with predominant N_2_O formation via the *cis* pathway, which is in agreement with the previous conclusions of Fehling and Friedrichs,^31^ Bringas et al.,^32^ and Zhang and Thynell.^33^

To the best of our knowledge, our isotopomer-selective measurement represents the first direct experimental proof of this hitherto mainly theoretical conclusion. In contrast to pH-dependent rate constant studies of the overall N_2_O formation and its mechanistic interpretation,^33^ the determination of the site preference turns out to be a better-suited indicator to identify the species directly decaying into N_2_O, here either the neutral *cis*-hyponitrous acid HONNOH (δ^15^N^SP^ ≈ 28‰) or the *cis*-HONNO^–^ anion (δ^15^N^SP^ ≈ 43‰). Significant N_2_O formation through the decomposition of the *trans*-HONNO^–^ anion would have resulted in a much lower site preference (δ^15^N^SP^ ≈ −7.5‰). Assuming (i) an initial *cis*/*trans* HNO dimer formation ratio of 164 to 500 (obtained either from the 12.6 kJ/mol free enthalpy difference of the activated complexes from the SMD/wB97XD/6-311++G** calculations of Zhang and Thynell^33^ or from the Kirkwood model estimate accounting for solute–solvent dipole–dipole interaction reported by Fehling and Friedrichs^31^) and (ii) a fully kinetically decoupled N_2_O formation from the *cis* and *trans* pathways, the *trans* contribution to the overall measured δ^15^N^SP^ is only about −(0.07–0.23)‰. This is well below the uncertainty of our theoretical predictions. Note that coupling of the *cis* and *trans* pathways through interconversion of the diverse intermediate species is hampered by high isomerization barriers stemming from the more or less double-bond character of the N–N bond. As outlined by Fehling and Friedrichs,^31^ even the lowest barrier of the *trans*-HON­(H)­NO ⇌ *cis*-HON­(H)­NO isomerization exhibits a free enthalpy of activation on the order of 73.4 kJ/mol and hence is very slow at room temperature. Moreover, the equilibrium is heavily on the side of the *cis* isomer, and the final N_2_O decomposition step from *trans*-HONNO^–^ is 2 and 6 orders of magnitude slower than the decomposition of *cis*-HONNOH and *cis*-HONNO^–^, respectively (see Tables 2, S5, and S6 in the Supporting Information). Therefore, even if equilibration between the *cis* and *trans* species would take place to some extent, the overall site preference would still be dominated by the *cis* decomposition.

Finally, next to the involvement of the *trans*-species, significant N_2_O formation from alternative decomposition steps that are not included in the equilibration model outlined in Figure 1 could challenge our straightforward interpretation. For example, in their careful analysis of the *cis* reaction system, Zhang and Thynell^33^ also considered the role of the protonation of *cis*-HONNOH under superacidic conditions. Actually, N_2_O formation according to the (experimentally yet unvalidated) reaction

$$cis‐\mathrm{HONNOH}+H_{2}O+H_{3}O^{+}\to N_{2}O+2H_{2}O+H_{3}O^{+}$$ 14

could indeed take place, but only under superacidic conditions with pH < 0.

### 5.2 pH Dependence of Site Preference

Figure 5 reveals a clear pH dependence of the site preference. Note that the lower panel of Figure 5 is merely an enlargement of the upper panel, focusing on *cis* decomposition. The strong pH dependence is related to the pH-dependent fraction of N_2_O resulting from *cis*-HONNOH and *cis*-HONNO^–^ decomposition. No pH dependence is seen for the *trans* pathway (upper panel) as N_2_O formation only takes place from *trans*-HONNO^–^. A p*K*_a_ of 5.4 has been assumed for the *cis*-HONNOH ⇌ *cis*-HONNO^–^ + H^+^ acid-base equilibrium. Therefore, by accounting for the rate constant ratio *k*_A^–^_/*k*_HA_ = *k*_ratio_ = 3.1 × 10^4^ (hence log­(*k*_A^–^_/*k*_HA_) = 4.5), the transition between preferential N_2_O formation from *cis*-HONNOH (at low pH) and *cis*-HONNO^–^ (at high pH) takes place at pH = 5.4 – 4.5 = 0.9. This is rather close to pH = 0.6 used in our N_2_O synthesis, hence the experimental site preference value is a weighted sum over the site preference of N_2_O from *cis*-HONNOH and *cis*-HONNO^–^ decomposition. For instance, when pH equals 0.6, the factor *k*_ratio_/(*k*_ratio_ + 10^–(pH^ ^–p*^K^*_a_)^) in eq 13 evaluates to 0.32, indicating that 32% of the generated N_2_O originates from *cis*-HONNO^–^ and the remaining 68% from *cis*-HONNOΗ decomposition. The blue dashed and green dash-dotted curves in the lower panel highlight the effect of the variation of p*K*_a_ by ±0.5 units and of the rate constant ratio by a factor of 2. The resulting shifts of the sigmoidal curves indicate that the predicted values for δ^15^N^SP^ depend very sensitively on the accuracy of these variables. As expected, with increasing p*K*_a_, the sigmoidal curve shifts to higher pH values, corresponding to a higher contribution of *cis*-hyponitrous acid decomposition. The same effect can be achieved by reducing the *k*_ratio_, corresponding to higher rate constants for the formation of N_2_O from *cis*-hyponitrous acid decomposition.

Moreover, we have found a significant effect of the tunneling model used to calculate the site preference. This is highlighted by the three curves in the upper and lower panels that correspond to three different scenarios labeled *a*, *b*, and *c*, where the dashes next to the labels in the lower plot indicate the low and high pH limits for δ^15^N^SP^ resulting from pure *cis*-HONNOH and *cis*-HONNO^–^ decomposition. Scenario *a* depicts a theoretical prediction without tunneling correction, *b* with Wigner, and *c* with Eckart tunneling. The difference between the Wigner and Eckart tunneling models is more pronounced for *cis*-HONNOH, going along with the much higher tunneling contribution of the rate constant values in the case of HONNOH (see Section 4.2). Whereas a simple Wigner correction provides a good approximation for weak tunneling, in general, Eckart tunneling is expected to yield a more reliable rate constant and with it a site preference estimate. However, in our case, the Eckart tunneling model prediction of δ^15^N_HA_^SP^ = 33.6‰ is already higher than the experimentally measured value. This remaining inconsistency is probably due to inaccuracies in the quantum chemical calculations (e.g., solvent model; see below) but could, in principle, also be due to our kinetic treatment within the all-equilibrium model or even unidentified N_2_O formation pathways. However, we consider the overall already good agreement between our experiments and the independent quantum chemically based prediction within a few per mille as confirmation of our approach. Note that a failure of the underlying assumption of fast equilibration of all relevant species would even shift the transition between the site preference values of *cis*-HONNOH and *cis*-HONNO^–^ to lower pH. This is because the *cis* dimerization pathway feeds into only the anionic species (see Figure 2). Hence, a slow acid-base equilibration would result in a shift of the site preference toward the higher *cis*-HONNO^–^ value.

In the following, we focus our discussion on the theoretical prediction based on the Wigner-corrected site preferences. This is not to say that we prefer the Wigner correction, but it simply accounts for the overall better agreement of the experimental data with this model. Clearly, future experiments with N_2_O syntheses performed at variable pH using buffer solutions are desirable to determine the exact course and to further validate the theoretically predicted sigmoidal shape of the pH-dependent total site preference, as well as to define both the dynamic range and the working limits of the proposed model.

Remembering our original claim to use HNO dimerization as a chemical reference standard, it could also be beneficial to work at even lower or sufficiently high pH to ensure a low pH dependence resulting from either pure *cis*-HONNOH or *cis*-HONNO^–^ decomposition. However, at too low pH, additional reaction pathways such as the one shown in eq 14 may prevail, and at too high pH, the acid-base equilibration may become too slow to compete with the very fast *cis*-HONNO^–^ decomposition. We therefore recommend carrying out such measurements under acidic but not superacidic conditions. In the end, it is only important that the corresponding site preference value is known accurately enough to serve as a reliable absolute reference standard.

### 5.3 *T* Dependence of Site Preference

Further verification of our overall model comes from the site preference measurements using N_2_O samples generated at variable synthesis temperatures. Figure 6 compares our temperature-dependent site preference data (black dots) to the theoretical predictions. The theoretical estimates are based on (i) the room temperature site preferences of δ^15^N_HA, *cis*_^SP^ = 27.7‰, δ^15^N_A^–^, *cis*_^SP^ = 43.0‰ (same as in the lower panel of Figure 5), and δ^15^N_A^–^, *trans*_^SP^ = −7.4‰, (ii) a room temperature reference p*K*_a_ of 5.4 (where a variation simply causes an offset in the predicted site preference, as highlighted by the dash-dotted blue curves), (iii) the temperature dependences of the decomposition rate constants (resulting from the higher activation energy for the *cis*-HONNOH decomposition); (iv) the theoretically predicted temperature dependence of the site preferences using Eckart tunneling factors, and (v) an assumed enthalpy of reaction of Δ_r_ *H* = 5 kJ/mol for the deprotonation step of the *cis* acid. According to Van’t Hoff’s equation, the latter determines the temperature-dependent equilibrium ratio of *cis*-HONNOH and *cis*-HONNO^–^. Δ_r_ *H* = 5 kJ/mol corresponds to a typical value for a weak acid. Note, however, that changing Δ_r_ *H* from −5 to 15 kJ/mol (green dashed curves, where the one with lower temperature dependence corresponds to the more positive reaction enthalpy) does not seriously deteriorate the very good agreement with the experimentally observed temperature trend. Instead, most of the temperature dependence results from the expected decreasing isotopic effects with increasing temperature, as well as the temperature dependence of the decomposition rates, favoring the lower δ^15^N_HA, *cis*_^SP^ values at higher temperature. Again, the experiments are fully in agreement with the formation of N_2_O from the *cis*-pathway. An opposite temperature trend would have been expected for the *trans*-pathway (dashed black curve in Figure 6).

### 5.4 Comparison with Literature Values

In Figures 5 and 6, our experimental site preference values (black dots) for N_2_O generation from HNO dimerization are compared with the available literature data. The red diamond in Figure 5 indicates the δ^15^N^SP^ = 30.9 ± 2.0‰ IRMS value from Toyoda et al.^15^ Nearly identical to this work, N_2_O was obtained from a nitrite reduction with trimethylamine-borane, but in a less acidic solution at pH = 1. The site preference has been obtained from the N_2_O fragmentation pattern by IRMS analysis with measured isotope ratios linked to the atmospheric N_2_ reference standard. The error bars indicate the 2σ standard deviation as determined from their original five data points listed in Table 2 of ref 15. Note that Toyoda et al. actually have reported a δ-based site preference value of SP = 30.1‰, which we have converted to δ^15^N^SP^ = 30.9‰ using their reported averaged δ^15^N^β^ = −25.9‰ value using eq 1. In their paper, the synthesis temperatures have not been specified, but most probably were close to room temperature. Although this data point seems to be in quantitative agreement with our measurement within error limits, provided that the pH dependence determined by us is reliable, a somewhat higher value would have been expected. Along those lines, assuming the pH dependence of the site preference resulting from scenarios a, *b*, and *c*, this may indicate a small systematic bias of the IRMS in the range of −1.2‰ to −2.5‰ compared to our direct measurement. Although such a small offset cannot be confirmed based on the current data accuracy, we note that it seems consistent with a previously observed systematic offset of about −4‰ from another IRMS study. Kantnerova et al.^13^ performed accurate site preference measurements on thermally equilibrated N_2_O samples. Their reported IRMS data (Table 2 and Figure 4b in ref 13, 200 °C equilibrated samples) are systematically lower by about 4‰ compared to the theoretical equilibrium ratio predictions taken from Wang et al.^12^

Another study has been performed and published as part of the PhD thesis of Fehling.^85^ His data are included in Figures 5 and 6 as red squares. Using a diode laser-based near-infrared cavity ringdown spectrometer,^42^ this study targeted a direct measurement of the site preference. Conceptually different from the present study, Fehling could not rely on HITRAN data but first determined accurate line strengths of selected rovibrational 3001 ← 0000 band transitions of the two isotopomers at wavenumbers of around 5926 cm^–1^. For line strength determination, almost pure isotopomer samples with enrichments >98% have been analyzed, where the isotopic composition of the sample gases was precisely determined by Fourier transform ion cyclotron resonance mass spectrometry (FT-ICR-MS) beforehand. Accounting for error propagation resulting from uncertainties in cell temperature, relative line strengths, and statistical error, an overall accuracy on the order of ±10‰ could be achieved. For N_2_O from a nitrite reduction with trimethylamine-borane at pH values ranging from 0.58 ≤ pH ≤ 0.66, δ^15^N^SP^ = 40 ± 10‰ has been reported. As seen from Figure 5, within the reported uncertainty range, their result is quite consistent with our data. What is more important at this point, however, is that these completely independent and direct measurements also provide a clear indication that N_2_O is predominantly formed via the *cis*-pathway. Temperature-dependent data from Fehling are listed in Figure 6. Although the precision of his data does not reveal any clear temperature dependence, they are essentially consistent with the new, more precise data from our comb measurements.

### 5.5 Quantum Chemistry Model and Solvent Effect

The overall very good agreement of the theoretically predicted site preference values with the experimental site preference data within a few per mille, as well as the minor influence of the selected basis set, should not hide the fact that the first-principle prediction can be subject to some systematic errors. First of all, it should be noted that the accuracy of theoretically determined δ^15^N^SP^ values is affected by the uncertainties in reported scaling factors of harmonic frequencies and ZPEs for a given level of theory. For example, a typical uncertainty of 2–5% in empirical scaling factors,^86^ translates to an uncertainty on the order of ∼1‰ for the predicted site preference. Moreover, treating solvation effects in terms of the IEFPCM polarization continuum solvent model may cause some uncertainties. In our approach, it has been implicitly assumed that explicit water molecules and the corresponding embedding of the neutral HONNOH and the HONNO^–^ anion into the surrounding hydrogen-bond network would not cause significant isotopic effects. To better assess the influence of the solvent, Table 5 compares δ^15^N^SP^ values obtained either with the IEFPCM solvent model or for pure gas phase calculations. With a site preference difference between liquid and gas phase, Δ­(δ^15^N_total_^SP^) = +8.5‰ for the anion and +0.4‰ for the neutral acid, the solvent effect on the total site preference is much more pronounced for the anionic species, where the main effect can be traced back to the differences in the KIE values. Interestingly, without accounting for the solvent, the predicted site preference difference between HONNOH and HONNO^–^ largely diminishes in the gas phase and would be difficult to detect. Clearly, solvent effects are important, which raises the question of to what extent explicit water molecules would affect theoretical δ^15^N^SP^ predictions? A detailed analysis of such subtle effects would have gone beyond the scope of this paper. Therefore, future studies should focus on investigating the influence of explicit water molecules in more detail, ideally accounting for the 10% dioxane content in the synthesis mixtures as well.

**Table 5:** Solvent Effect^a^

	*cis*-hyponitrite anion		*cis*-hyponitrous acid	
	liquid	gas	Liquid	gas
δ^15^N_KIE_^SP^/‰	33.6	23.7	30.2	26.9
δ^15^N_EIE_^SP^/‰	9.6	11.0	3.1	6.0
$\delta^{15}N_{\mathrm{total}}^{\mathrm{SP}}$ /‰	43.5	35.0	33.6	33.2

a Comparison of calculated intrinsic isotope effects (B3LYP/aug-cc-pVTZ, ISOEFF, and Eckart tunneling) in the gas phase and in liquid water (IEFPCM). The total SP values are calculated using the exact forms of eqs 8 and 10.

### 5.6 Frequency Comb Approach

A principal advantage of our broadband frequency-comb approach lies in the fact that multiple line pairs can be analyzed at once with high spectral precision. This reduces fit uncertainties compared with narrow-bandwidth CW laser-based methods, which typically measure only a single pair of lines. The measurement of several line pairs not only improves the precision but also facilitates the detection of possible cross-sensitivities with other species in the gas mixture. These can interfere with individual absorption lines, where multiple measuring points make it easier to recognize possible outliers. Another advantage of broadband detection is that it allows the selection of line pairs that share the rotational quantum number and hence have very similar lower state term energies. In contrast, in methods based on narrow-tuning-range light sources, such as CW diode lasers, line pairs with lower state energy differences of up to hundreds of cm^–1^ need to be selected, causing a considerable temperature-sensitivity of the determined site preference value. Based on relative Boltzmann population differences, δ^15^N^SP^ can be estimated to change by (Δ*E*/*k*_B_ *T*) × (Δ*T*/*T*) under nonstabilized temperature conditions. For example, for a difference in the lower state energy of 200 cm^–1^ and assuming a temperature change of 1 K around 297 K, a change in δ^15^N^SP^ by 3.3‰ can be estimated. In this work, the difference in the lower state term values (Δ*E* = *E*_α_^″^ – *E*_β_^″^) between the two isotopomers was very small, ranging from 0.17 to 14 cm^–1^ (see Table S12 in the Supporting Information).

This work aimed to provide an absolute, accurate value of the N_2_O site preference of a promising chemical reference standard material by measuring multiple line pairs rather than achieving the best possible precision for the intensity ratio of a single line pair or the best possible detection sensitivity. Future improvements of the comb-based spectrometer in terms of sensitivity are straightforward, for example, by implementation of a multipass approach or by measuring the 2 orders of magnitude stronger ν_1_ fundamental band around 2200 cm^–1^. In this context, it should also be mentioned that modern sophisticated mid-IR diode laser-based instruments are already available for N_2_O isotopomer ratio measurements that rely on single line pair analysis using tunable direct absorption spectroscopy (TDAS), cavity ringdown spectroscopy (CRDS), and off-axis integrated cavity output spectroscopy (OA-ICOS).^46^ Typically, such instruments offer a precision for δ^15^N^SP^ measurement on a 1‰ level with moderate averaging times of several minutes and are suitable for the analysis of ambient samples with N_2_O mixing ratios on the order of several hundreds of ppb. However, as of yet, such instruments rely on frequent measurement of reference standard gas samples to overcome instrumental drift and to link the measured value to a reference scale.

## 6 Conclusions

Previous work suggested that initial HNO dimerization in solution takes place through the *cis* dimer, followed by a sequence of isomerization and protonation/deprotonation steps.^31–33^ Here, we used high-precision, broadband optical frequency comb spectroscopy in the mid-infrared spectral region to measure isotopomerically labeled N_2_O generated from the HNO dimerization reaction to validate this hypothesis. Simultaneous measurements of almost equal abundances of the resulting isotopocules allowed us to identify 30 pairs of spectrally resolved absorption lines for the α and β isotopomers. In combination with the line strength parameters from the HITRAN2020 database,^80^ we determined the ^15^N-site preference (δ^15^N^SP^) with high precision and accuracy. The experiments have been complemented with quantum-chemical and transition state theory calculations of energies and rate constants to derive an alternative and independent prediction of the absolute site preference based on first principles. A detailed kinetic analysis of the complex reaction pathways and equilibria revealed that the site preference is determined by fast equilibration of the *cis*-hyponitrite anion and *cis*-hyponitrous acid, along with simultaneous decomposition of both species. The overall site preference is a result of these two competing pathways and hence is strongly dependent on the pH set during synthesis. Relying on our rate constant estimates and the most recent theoretically derived p*K*_a_ value of *cis*-hyponitrous acid,^33^ the transition between a dominant *cis*-hyponitrous acid and *cis*-hyponitrite decomposition takes place at pH ≈ 1. On the one hand, the overall very good agreement between the theoretical predictions and the experimental results can be seen as direct evidence for a dominant N_2_O formation via the *cis*-decomposition pathway. On the other hand, our results demonstrate that HNO dimerization under acidic conditions can indeed serve as a suitable and transferable chemical reference standard for absolute N_2_O site preference determination. These promising results, both in terms of frequency comb-based isotopic analysis and absolute δ^15^N^SP^ determination, call for future work. This includes, among others, linking our δ^15^N^SP^ ratio-based measurements to the more common δ-based *SP* values. These are typically referenced to N_2_O from thermal decomposition of ammonium nitrate (NH_4_NO_3_)^10,87^ or tropospheric N_2_O, the latter found to exhibit very stable “steady-state” SP values despite the large increase in anthropogenic N_2_O emissions.^1,88^ The comparison of our results with that of Toyoda et al.^15^ in Figure 5 suggests a small offset of about 2‰ between our and Toyoda’s scale, which is within the error limits but would be in agreement with a previously observed offset of about 4‰, as deduced from a comparison of the mass spectrometric data of Kantnerova et al.^13^ with theoretical predictions for their thermally equilibrated N_2_O samples. Clearly, an intercomparison study involving thermally equilibrated N_2_O, N_2_O derived from HNO dimerization, N_2_O from the decomposition of NH_4_NO_3_ (to correlate the absolute measurements with the Toyoda site preference scale), and tropospheric N_2_O (used as working reference in many laboratories) utilizing mass spectrometry, QCLAS, and our frequency comb technique would be highly advantageous for refining and validating current methodologies, as well as for setting N_2_O site preference measurements on an absolute scale. The availability of absolute and accurate δ^15^N^SP^ values is a prerequisite for fully exploiting the potential of theoretically predicted N_2_O site preferences for elucidating reaction mechanisms. The already satisfying agreement between our ab initio predictions and experimental values for HNO dimerization points in this direction. However, prior to achieving this goal, the experimental determination of the pH-dependent site preference could and should serve as a validation of the kinetic model and the absolute site preference values. Moreover, further refined theoretical studies are needed to better account for subtle solvent effects known to influence the isotopic signature. These should focus on more advanced solvation models, particularly those incorporating explicit water molecules, to investigate potential isotopic effects. Such a combined experimental and theoretical approach is expected to further reduce the remaining δ^15^N^SP^ (N_2_O) site preference differences between first-principle predictions and precise spectroscopic measurements made possible by the comb-based approach.

## Supplementary Material



## Notes

Site preference terminology; Kinetic scheme for total δ^15^N^SP^ total determination; Calculated rate constants at selected temperatures; δ^15^N^SP^ of the trans-pathway; Basis set dependence; Selected line pairs and line pair intensity ratios from comb spectroscopy (PDF)

## Notes

Dedicated to Dr. Carsten Fehling, whose passion for science continues to inspire us.
