# Supplementary material for: HNO Dimerization as a Chemical Reference Standard for N2O Isotopomer Ratio: Ab Initio Calculations, Formation Kinetics, and Frequency Comb Spectroscopy
Source: J Am Chem Soc. 2025 Oct 10;147(42):38110–27. doi: 10.1021/jacs.5c09983 (PMC12577524; doi:10.1021/jacs.5c09983)
Supplement: Supplementary file 1 [file ja5c09983_si_001.pdf]

Supporting Information

HNO Dimerization as a Chemical Reference Standard for N_2O Isotopomer Ratio: Ab initio Calculations, Formation Kinetics, and Frequency Comb Spectroscopy

Ibrahim Sadiek*,[1,2] Adrian Hjältén,[1] Gernot Friedrichs*,[3,4] Aleksandra Foltynowicz[1]

[1]*Department of Physics, Umeå University, 901 87, Umeå, Sweden*

[2]*Experimental Physics V, Faculty of Physics and Astronomy, Ruhr University Bochum, 44780 Bochum, Germany*

[3]*Institute of Physical Chemistry, Christian-Albrechts-University Kiel, 24118 Kiel, Germany*

[4]*Kiel Marine Science-Centre for Interdisciplinary Marine Sciences, Christian-Albrechts-University Kiel, 24098 Kiel, Germany*

Dedicated to Dr. Carsten Fehling whose passion for science continues to inspire us.

Contents

A. Site Preference Terminology

(i) Used Terminology
(ii) $SP = \delta^{15}\mathrm{N}^{\mathrm{SP}}$ limit.
(iii) SP to $\delta^{15}\mathrm{N}^{\mathrm{SP}}$ conversion.

B. Kinetic Scheme for Total $\delta^{15}\mathrm{N}^{\mathrm{SP}}_{\mathrm{total}}$ Determination

Figure S 1: Reaction diagram for the cis-pathway of HNO dimerization.
(i) Interconversion and decomposition of HONNO^-.
(ii) Interconversion and decomposition of HONNOH.
(iii) Acid-base equilibrium between hyponitrite and hyponitrous acid.
(iv) Influence of non-hydrogen-bonded symmetric form of HONNOH.
(v) ^{15}N-site preference of $trans$-HONNO^- decomposition.
Figure S 2: Reaction diagram for the $trans$-pathway of HNO dimerization.

C. Calculated Rate Constants at Selected Temperatures

Table S 1: Interconversion and decomposition of HONNO^- (α).
Table S 2: Interconversion and decomposition of HONNO^- (β).
Table S 3: Interconversion and decomposition of HONNOH (α).
Table S 4: Interconversion and decomposition of HONNOH (β).

D. $\mathtt{trans}$-Pathway Data

Table S 5: Rate Constants of $trans$-HONNO^- decomposition (inwards OH).
Table S 6: Rate Constants of $trans$-HONNO^- decomposition (outwards OH).
Table S 7: (Relative) Free Enthalpies of $trans$-HONNO^-.
Table S 8: $\delta^{15}\mathrm{N}_{\mathrm{total},trans}$ of $trans$-HONNO^- decomposition.

E. Basis Set Dependence

Table S 9: Comparison of $\delta^{15}\mathrm{N}^{\mathrm{SP}}$ values.
Table S 10: Comparison of calculated electronic energies.
Table S 11: Comparison of calculated imaginary frequencies.

F. Selected Line Pairs from Comb Spectroscopy

Table S 12: Line List and $\alpha - \beta$ Term Value Differences.
Figure S 3: Statistics of fitted relative line intensity ratios.

A. Site Preference Terminology

(i) Used Terminology

– Isotope ratios:

$$R_\alpha = \frac{[^{14}\mathrm{N}^{15}\mathrm{NO}]}{[^{14}\mathrm{N}^{14}\mathrm{NO}]} \quad , \quad R_\beta = \frac{[^{15}\mathrm{N}^{14}\mathrm{NO}]}{[^{14}\mathrm{N}^{14}\mathrm{NO}]} \quad , \quad R_{\mathrm{N}_2} = \frac{[^{15}\mathrm{N}]}{[^{14}\mathrm{N}]}$$

– δ values:

$$\delta^{15}\mathrm{N}^\alpha = \left(\frac{R_\alpha}{R_{\text{reference}}} - 1\right) \cdot 1000\ ‰ \quad , \quad \delta^{15}\mathrm{N}^\beta = \left(\frac{R_\beta}{R_{\text{reference}}} - 1\right) \cdot 1000\ ‰$$

Here, $R_{\text{reference}}$ is the isotope ratio of the common reference standard, i.e. $R_{\mathrm{Air-N_2}} = 0.0036765$.

–'Ratio-based' site preference (used in this work, preferred in theoretical isotope modeling and spectroscopy):

$$\delta^{15}\mathrm{N}^{\mathrm{SP}} = \left(\frac{R_\alpha}{R_\beta} - 1\right) \times 1000\ ‰ = \left(\frac{[^{14}\mathrm{N}^{15}\mathrm{NO}]}{[^{15}\mathrm{N}^{14}\mathrm{NO}]} - 1\right) \times 1000\,‰$$

– 'δ-based' site preference (established in MS community, common in N_2O site preference literature):

$$SP = \delta^{15}\mathrm{N}^\alpha - \delta^{15}\mathrm{N}^\beta$$

(ii) $SP = \delta^{15}\mathrm{N}^{\mathrm{SP}}$ limit

For $R_\alpha/R_{\text{reference}} \approx 1$, $R_\beta/R_{\text{reference}} \approx 1$, and $[^{14}\mathrm{N}^{15}\mathrm{NO}]/[^{15}\mathrm{N}^{14}\mathrm{NO}] \approx 1$, the two alternative definitions for the site preference become equal. Using the Taylor expansion of $\ln x$ around 1 for $x \approx 1$,

$$\ln x = (x-1) - \frac{(x-1)^2}{2} + \ldots \approx x - 1\,,$$

it holds:

$$\frac{SP}{1000\,‰} = \frac{\delta^{15}\mathrm{N}^\alpha - \delta^{15}\mathrm{N}^\beta}{1000\,‰} = \left(\frac{R_\alpha}{R_{\text{reference}}} - 1\right) - \left(\frac{R_\beta}{R_{\text{reference}}} - 1\right)$$
$$\approx \ln\frac{R_\alpha}{R_{\text{reference}}} - \ln\frac{R_\beta}{R_{\text{reference}}} = \ln\frac{R_\alpha}{R_\beta} \approx \frac{R_\alpha}{R_\beta} - 1 = \frac{\delta^{15}\mathrm{N}^{\mathrm{SP}}}{1000\,‰}$$

(iii) SP to $\delta^{15}\mathrm{N}^{\mathrm{SP}}$ conversion

$$\begin{aligned}
\delta^{15}\mathrm{N}^{\mathrm{SP}} &= \left(\frac{R_\alpha}{R_\beta} - 1\right) \times 1000\ ‰ \\
&= \left(\frac{R_\alpha/R_{\text{reference}} - R_\beta/R_{\text{reference}}}{R_\beta/R_{\text{reference}}}\right) \times 1000\ ‰ \\
&= \frac{(R_\alpha/R_{\text{reference}} - 1) \times 1000\,‰ - (R_\beta/R_{\text{reference}} - 1) \times 1000\,‰}{1 + (R_\beta/R_{\text{reference}} - 1)} \\
&= \frac{(\delta^{15}\mathrm{N}^\alpha - \delta^{15}\mathrm{N}^\beta) \times 1000\,‰}{1000\,‰ + \delta^{15}\mathrm{N}^\beta} = \frac{SP \times 1000\,‰}{1000\,‰ + \delta^{15}\mathrm{N}^\beta} \\
\delta^{15}\mathrm{N}^{\mathrm{SP}} &= \frac{SP}{1 + \delta^{15}\mathrm{N}^\beta/1000\,‰}
\end{aligned}$$

Example: Toyoda et al. (*Soil Biology & Biochemistry* 37 (2005) 1535-1545) report $SP = 30.1\,‰$ for N_2O from nitrite reduction. This value is based on $\delta^{15}N^{\alpha} = 4.2\,‰$ and $\delta^{15}N^{\beta} = -25.9\,‰$ (average of 5 data points in Table 2 of their paper), referenced to $R_{\text{Air}-N_2}$. Consequently, their δ-based SP value corresponds to $\delta^{15}N^{\text{SP}} = 30.1\,‰/(1 - 25.9\,‰/1000\,‰) = 30.9\,‰$. The difference of $0.8\,‰$ is significant and needs to be taken into account for the direct comparison of their reported δ-based SP and our ratio-based $\delta^{15}N^{\text{SP}}$ (see Fig. 5 in main paper). Note that the necessary offset varies depending on the used reference standard for SP determination and the site preference value itself. Therefore, in terms of reporting absolute site preference values, the use of ratio-based $\delta^{15}N^{\text{SP}}$ is advantageous.

B. Kinetic Scheme for Total $\delta^{15}N^{\text{SP}}_{\text{total}}$ Determination

In order to obtain an analytical expression for the overall site preference $\delta^{15}N^{\text{SP}}_{\text{total}}$ from the *cis*-pathway, it is essential to consider both the kinetic isotope effects (KIE) and equilibrium isotope effects (EIE) relevant to the intermediates hyponitrite anion ($HONNO^-$, A^-) and hyponitrous acid (HONNOH, HA). As shown in the Figure S1 below, initial dimerization of two HNO molecules forms the symmetric *cis*-ON(H)N(H)O, followed by several protonation and deprotonation steps yielding $HONNO^-$. The key reactions to describe the $\delta^{15}N^{\text{SP}}_{\text{total}}$ site preference in the generated N_2O include the very fast isotopomer interconversion and decomposition of either (*i*) hydrogen-bridged cyclic $HONNO^-$ or (*ii*) HONNOH intermediates as well as (*iii*) their mutual acid-base equilibrium, including (*iv*) the formation of the non-hydrogen-bridged symmetric (open) *sym*-HONNOH. Note that the non-hydrogen-bridged symmetric form of $HONNO^-$, which can be directly protonated to *sym*-HONNOH, exists as well. It is excluded from the Figure as it is energetically unfavorable and therefore is only present at very low concentrations and does not need to be taken into account for the calculation of $\delta^{15}N^{\text{SP}}_{\text{total}}$. However, it also contributes to the rapid equilibration of the intermediates that is assumed to be fast in the following.

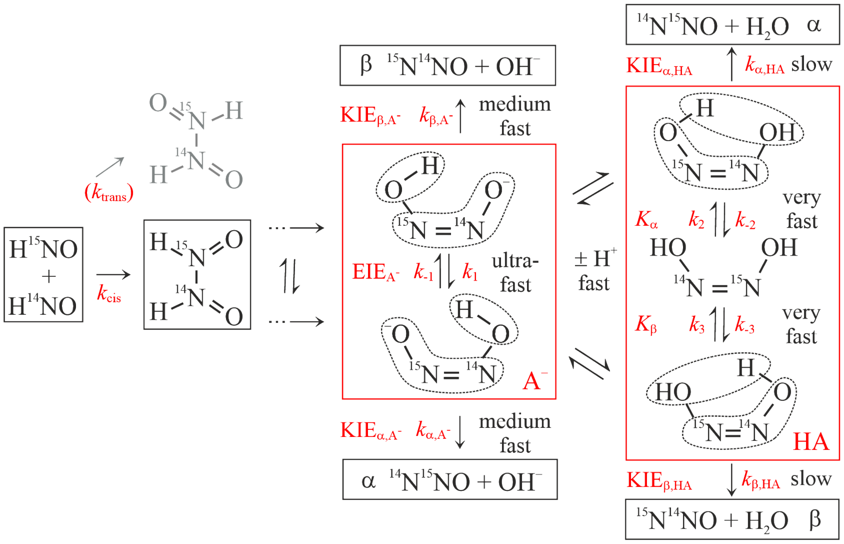

Figure S1: Reaction diagram for the *cis*-pathway of HNO dimerization.

(i) Interconversion and decomposition of $HONNO^-$

We first consider the rapid interconversion between the α and β isotopomers of the hyponitrite anion (A^-), defined by rate coefficients k_1 and k_{-1}, and their subsequent decomposition with rate coefficients k_{α,A^-} and k_{β,A^-} for the α and β pathways, respectively.

$$^{15}N^{14}NO + OH^- \xleftarrow{k_{\beta,A^-}} HO^{15}N^{14}NO^- \underset{k_{-1}}{\overset{k_1}{\rightleftharpoons}} HO^{14}N^{15}NO^- \xrightarrow{k_{\alpha,A^-}} {}^{14}N^{15}NO + OH^- \tag{S1}$$

The corresponding reaction coordinate diagram is shown in Figure 2 of the main paper. For the α and β pathways, we have:

$$\frac{\mathrm{d}\left[^{14}N^{15}NO\right]}{\mathrm{d}t} = k_{\alpha,A^-}\left[HO^{14}N^{15}NO^-\right] \tag{S2.1}$$

$$\frac{\mathrm{d}\left[^{15}N^{14}NO\right]}{\mathrm{d}t} = k_{\beta,A^-}\left[HO^{15}N^{14}NO^-\right] \tag{S2.2}$$

At reaction time t, the ratio of the concentrations of the α and β N_2O product becomes:

$$\frac{\left[^{14}N^{15}NO\right]}{\left[^{15}N^{14}NO\right]} = \frac{k_{\alpha,A^-}}{k_{\beta,A^-}} \times \frac{\int_0^t \left[HO^{14}N^{15}NO^-\right]\mathrm{d}t}{\int_0^t \left[HO^{15}N^{14}NO^-\right]\mathrm{d}t} \tag{S3}$$

As the equilibration of the two isotopomeric species of $HONNO^-$ is very fast compared to their decomposition rates forming N_2O, the respective concentration ratio remains in thermodynamic equilibrium:

$$\frac{\left[HO^{14}N^{15}NO^-\right]}{\left[HO^{15}N^{14}NO^-\right]} = \frac{k_1}{k_{-1}} \tag{S4}$$

When substituting $\left[HO^{14}N^{15}NO^-\right]$ from Eq. S4 into Eq. S3, the integrals cancel out for any time (including for the final α/β ratio at $t \to \infty$) and it holds:

$$\frac{\left[^{14}N^{15}NO\right]}{\left[^{15}N^{14}NO\right]} = \frac{k_{\alpha,A^-}}{k_{\beta,A^-}} \times \frac{k_1}{k_{-1}} = \frac{k_{\alpha,A^-}}{k_{1414,A^-}}\frac{k_{1414,A^-}}{k_{\beta,A^-}} \times \frac{k_1}{k_{-1}} = \frac{\mathrm{KIE}_{\alpha,A^-}}{\mathrm{KIE}_{\beta,A^-}} \cdot \mathrm{EIE}_{A^-} \tag{S5}$$

Here, k_{1414,A^-} represents the decomposition rate constant of the $HO^{14}N^{14}NO^-$ species. Eq. S5 clearly shows that the ratio of $^{14}N^{15}NO$ and $^{15}N^{14}NO$ can be expressed in terms of the kinetic isotope effects $\mathrm{KIE}_{\alpha,A^-}$ and KIE_{β,A^-} as well as the equilibrium isotope effect EIE_{A^-} associated with the interconversion of the α and β isotopomers. In terms of the ^{15}N site preference $\delta^{15}N^{SP}_{A^-}$, and with $\mathrm{KIE}_{\alpha,A^-}$, KIE_{β,A^-}, and EIE_{A^-} all close to one, Eq. S5 can be rewritten as:

$$\delta^{15}N^{SP}_{A^-} = \frac{\left[^{14}N^{15}NO\right]}{\left[^{15}N^{14}NO\right]} - 1 = \frac{\mathrm{KIE}_{\alpha,A^-}}{\mathrm{KIE}_{\beta,A^-}} \cdot \mathrm{EIE}_{A^-} - 1 \approx \delta^{15}N^{\alpha,A^-}_{KIE} - \delta^{15}N^{\beta,A^-}_{KIE} + \delta^{15}N^{SP}_{\mathrm{EIE}_{A^-}} \tag{S6}$$

(ii) Interconversion and decomposition of HONNOH

The potential energy surface of hyponitrous acid decomposition involves a central non-hydrogen-bonded symmetric (open) conformer *sym*-HONNOH, which is thermodynamically more stable than the hydrogen-bonded isotopomers. The corresponding reaction coordinate diagram is shown in Figure 2 of the main paper. Therefore, the interconversion between the α or β species and the total EIE_{HA} is determined by the double equlibrium:

$$\begin{aligned} {}^{15}N^{14}NO + H_2O &\xleftarrow{k_{\beta,HA}} \mathbf{HO}^{14}N^{15}N\mathbf{OH} \underset{k_3}{\overset{k_{-3}}{\rightleftharpoons}} sym\text{-}HO^{15}N^{14}NOH \\ &\underset{k_2}{\overset{k_{-2}}{\rightleftharpoons}} \mathbf{HO}^{15}N^{14}N\mathbf{OH} \xrightarrow{k_{\alpha,HA}} {}^{14}N^{15}NO + H_2O \end{aligned} \tag{S7}$$

Here, the bold printed H atom and OH group form the product water molecule (see Figure S1). As the equilibration of the two isotopomeric species of HONNOH is fast compared to decomposition, the respective concentration ratio in thermodynamic equilibrium is given by:

$$\frac{[\mathbf{H}\mathrm{O}^{15}\mathrm{N}^{14}\mathrm{N}\mathbf{OH}]}{[\mathbf{H}\mathrm{O}^{14}\mathrm{N}^{15}\mathrm{N}\mathbf{OH}]} = K_2 \cdot K_3 = \frac{k_2}{k_{-2}} \cdot \frac{k_3}{k_{-3}} = \mathrm{EIE_{HA}} \tag{S8}$$

Just like for the derivation of the $HONNO^-$ case, it follows for the corresponding site preference $\delta^{15}\mathrm{N}^{\mathrm{SP}}_{\mathrm{HA}}$:

$$\delta^{15}\mathrm{N}^{\mathrm{SP}}_{\mathrm{HA}} = \frac{\mathrm{KIE}_{\alpha,\mathrm{HA}}}{\mathrm{KIE}_{\beta,\mathrm{HA}}} \cdot \mathrm{EIE_{HA}} - 1 \approx \delta^{15}\mathrm{N}^{\alpha,\mathrm{HA}}_{\mathrm{KIE}} - \delta^{15}\mathrm{N}^{\beta,\mathrm{HA}}_{\mathrm{KIE}} + \delta^{15}\mathrm{N}^{\mathrm{SP}}_{\mathrm{EIE_{HA}}} \tag{S9}$$

(iii) Acid-base equilibrium between hyponitrite and hyponitrous acid

The overall site preference $\delta^{15}\mathrm{N}^{\mathrm{SP}}_{\mathrm{total}}$ can be expressed as a weighted average of the contributions of hyponitrite (A^-) and hyponitrous acid (HA) decomposition. The overall N_2O formation rate and instantaneous N_2O site preference is given by:

$$\left(\frac{\mathrm{d}[\mathrm{N_2O}]}{\mathrm{d}t}\right)_{\mathrm{total}} = \left(\frac{\mathrm{d}[\mathrm{N_2O}]}{\mathrm{d}t}\right)_{\mathrm{A^-}} + \left(\frac{\mathrm{d}[\mathrm{N_2O}]}{\mathrm{d}t}\right)_{\mathrm{HA}} = k_{\mathrm{A^-}}[\mathrm{A^-}] + k_{\mathrm{HA}}[\mathrm{HA}]_{\mathrm{eff}} \tag{S10}$$

$$\delta^{15}\mathrm{N}^{\mathrm{SP}}_{\mathrm{total}}(t) = R \cdot \delta^{15}\mathrm{N}^{\mathrm{SP}}_{\mathrm{A^-}} + (1-R) \cdot \delta^{15}\mathrm{N}^{\mathrm{SP}}_{\mathrm{HA}} \tag{S11}$$

The ratios R and $(1-R)$ correspond to the shares of N_2O formation through the hyponitrite and hyponitrous acid pathways:

$$R = \frac{k_{\mathrm{A^-}}\,[\mathrm{A^-}]_t}{k_{\mathrm{A^-}}\,[\mathrm{A^-}]_t + k_{\mathrm{HA}}\,[\mathrm{HA}]_{\mathrm{eff},t}} = \frac{k_{\mathrm{A^-}}([\mathrm{A^-}]/[\mathrm{HA}]_{\mathrm{eff}})_t}{k_{\mathrm{A^-}}([\mathrm{A^-}]/[\mathrm{HA}]_{\mathrm{eff}})_t + k_{\mathrm{HA}}} \tag{S12}$$

Here, $[\mathrm{HA}]_{\mathrm{eff}} = [\mathbf{H}\mathrm{O}^{15}\mathrm{N}^{14}\mathrm{N}\mathbf{OH}] + [\mathbf{H}\mathrm{O}^{14}\mathrm{N}^{15}\mathrm{N}\mathbf{OH}]$ and $[\mathrm{A^-}] = [\mathrm{HO^{14}N^{15}NO^-}] + [\mathrm{HO^{15}N^{14}NO^-}]$ has been used, where $[\mathrm{HA}]_{\mathrm{eff}}$ does not include the non-hydrogen-bonded symmetric (open) form of HONNOH (see Figure S1). Next to the acid/base equilibrium, also the isotopomer equilibria are fast compared to N_2O formation from the decomposition steps. The corresponding rate constants can be identified with $k_{\mathrm{A^-}} = (k_{\beta,\mathrm{A^-}} + k_{\beta\mathrm{A^-}})/2$ and $k_{\mathrm{HA}} = (k_{\alpha,\mathrm{HA}} + k_{\beta,\mathrm{HA}})/2$ to a very good approximation. Moreover, at constant pH, the ratio $[\mathrm{A^-}]/[\mathrm{HA}]_{\mathrm{eff}}$ is constant and R becomes a time-independent quantity. Hence, $\delta^{15}\mathrm{N}^{\mathrm{SP}}_{\mathrm{total}}$ remains unchanged throughout the whole reaction and the final time-integrated observed site preference does not depend on subtleties of the formation kinetics of the hyponitrite anion. Note that due to the final fast interconversion and acid-base equilibria of hyponitrite and hyponitrous acid, possible isotopomer preferences of preceding reaction steps are all overwritten and therefore do not need to be taken into account. However, $\delta^{15}\mathrm{N}^{\mathrm{SP}}_{\mathrm{total}}$ depends on the reaction conditions (pH, T), where the acid-base ratios $[\mathrm{A^-}]/[\mathrm{HA}]_{\mathrm{eff}}$ and $[\mathrm{A^-}]/[\mathrm{HA}]$ are given by the well-known Henderson-Hasselbalch equation:

$$\mathrm{pH} = \mathrm{p}K_{\mathrm{a}} + \log\left(\frac{[\mathrm{A^-}]}{[\mathrm{HA}]_{\mathrm{eff}}}\right) = \mathrm{p}K_{\mathrm{a,eff}} + \log\left(\frac{[\mathrm{A^-}]}{[\mathrm{HA}]}\right) \tag{S13.1}$$

$$\frac{[\mathrm{A^-}]}{[\mathrm{HA}]_{\mathrm{eff}}} = 10^{(\mathrm{pH}-\mathrm{p}K_{\mathrm{a}})} \tag{S13.2}$$

An effective $\mathrm{p}K_{\mathrm{a,\,eff}}$ value could be used to account for the equilibration of the hydrogen-bonded cyclic HONNOH with its symmetric (open) form. However, as it is further outlined in section (iv), the $\mathrm{p}K_{\mathrm{a}}$ value of the acid-base equilibrium $HONNO^- \rightleftharpoons$ *cyclic*-HONNOH can be directly used to calculate the site preference of the generated N_2O. Inserting Eq. S13.2 into Eq. S12 yields

$$R = \frac{k_{\mathrm{A}^-}}{k_{\mathrm{A}^-} + k_{\mathrm{HA}} \cdot 10^{(\mathrm{p}K_\mathrm{a} - \mathrm{pH})}} \tag{S14}$$

and from Eq. S11:

$$\delta^{15}\mathrm{N}^{\mathrm{SP}}_{\mathrm{total}} = \left(\delta^{15}\mathrm{N}^{\mathrm{SP}}_{\mathrm{A}^-} \cdot k_{\mathrm{A}^-} + \delta^{15}\mathrm{N}^{\mathrm{SP}}_{\mathrm{HA}} \cdot k_{\mathrm{HA}} \cdot 10^{(\mathrm{p}K_\mathrm{a} - \mathrm{pH})}\right) \cdot \frac{1}{k_{\mathrm{A}^-} + k_{\mathrm{HA}} \cdot 10^{(\mathrm{p}K_\mathrm{a} - \mathrm{pH})}} \tag{S15}$$

It is useful to expand this fraction with k_{HA} in order to express the final analytical expression for the total $\delta^{15}\mathrm{N}^{\mathrm{SP}}_{\mathrm{total}}$ in terms of the rate constant ratio $k_{\mathrm{ratio}} = k_{\mathrm{A}^-}/k_{\mathrm{HA}}$:

$$\delta^{15}\mathrm{N}^{\mathrm{SP}}_{\mathrm{total}} = \left(\delta^{15}\mathrm{N}^{\mathrm{SP}}_{\mathrm{A}^-} \cdot k_{\mathrm{ratio}} + \delta^{15}\mathrm{N}^{\mathrm{SP}}_{\mathrm{HA}} \cdot 10^{(\mathrm{p}K_\mathrm{a} - \mathrm{pH})}\right) \cdot \frac{1}{k_{\mathrm{ratio}} + 10^{(\mathrm{p}K_\mathrm{a} - \mathrm{pH})}} \tag{S16}$$

Equation Eq. S16 has been used to predict the ^{15}N-site preference for HNO dimerization at a given pH from theoretically calculated rate constants and the pK_a value of the acid-base pair *cis*-hyponitrite/*cis*-hyponitrous acid. The temperature dependence of $\delta^{15}\mathrm{N}^{\mathrm{SP}}_{\mathrm{total}}$ can be traced back to the temperature dependence of the rate constants and of the pK_a value, whereby the latter can be described by the Van't Hoff equation if the corresponding reaction enthalpy is known.

(iv) Influence of non-hydrogen-bonded *sym*-HONNOH

The pK_a value of the HONNOH/HONNO$^-$ acid-base pair is typically calculated for the hydrogen-bonded cyclic conformers. Whereas the thermodynamically favorable *cyclic* HONNO$^-$ completely predominates ($\Delta G \approx 21$ kJ/mol, corresponding to an equilibrium ratio [*open*-HONNO$^-$]/[*cyclic*-HONNO$^-$] $\approx 2 \times 10^{-4}$ at room temperature), the equilibrium is on the side of the symmetric (open) form (*sym*-HONNOH). Including the symmetry factor 2, $\Delta G = (-2.6 + RT \ln 2)$ kJ/mol, with ΔG(sym - cyclic) $= -2.6$, yields an equilibrium ratio [*sym*-HONNOH]/[*cyclic*-HONNOH] $= 1.43$ at room temperature. The existence of the *sym*-HONNOH intermediate shifts the acid-base equilibrium $\mathrm{A}^- + \mathrm{H}^+ \rightleftharpoons \mathrm{HA}$, with $[\mathrm{A}^-]$ = [*cyclic*-HONNO$^-$] and [HA] = [*cyclic*-HONNOH] + [*sym*-HONNOH], to the acid side. This can be best described by an effective acid constant p$K_{\mathrm{a,eff}}$ determined as follows.

By taking into account both α and β isotopomers, the equilibrium concentrations are given by the Henderson-Hasselbalch equation:

$$[\mathrm{HO^{14}N^{15}NO^-}] = [\textit{cyclic}\text{-}\mathbf{H}\mathrm{O^{15}N^{14}N}\mathbf{OH}] \cdot 10^{\mathrm{pH} - \mathrm{p}K_{\mathrm{a},\alpha}} \tag{S17.1}$$

$$[\mathrm{HO^{15}N^{14}NO^-}] = [\textit{cyclic}\text{-}\mathbf{H}\mathrm{O^{14}N^{15}N}\mathbf{OH}] \cdot 10^{\mathrm{pH} - \mathrm{p}K_{\mathrm{a},\beta}} \tag{S17.2}$$

For calculating the $[\mathrm{A}^-]/[\mathrm{HA}]$ ratio, it is sufficient to assume a common pK_a value, to set [*cyclic*-$\mathbf{H}$O^{15}N^{14}N$\mathbf{OH}$] = [*cyclic*-$\mathbf{H}$O^{14}N^{15}N$\mathbf{OH}$] = [*cyclic*-HONNOH]/2, and to assume a common equilibrium constant $K = (K_\alpha + K_\beta)/2$ for the equilibration of the *cyclic* isotopomers with the symmetric (open) conformer:

$$\frac{[\textit{sym}\text{-HONNOH}]}{[\textit{cyclic}\text{-}\mathbf{H}\mathrm{O^{15}N^{14}N}\mathbf{OH}]} \approx \frac{[\textit{sym}\text{-HONNOH}]}{[\textit{cyclic}\text{-}\mathbf{H}\mathrm{O^{14}N^{15}N}\mathbf{OH}]} = \frac{[\textit{sym}\text{-HONNOH}]}{[\textit{cyclic}\text{-HONNOH}]/2} = K \tag{S18}$$

Using these simplifications, on the one hand the $[\mathrm{A}^-]/[\mathrm{HA}]$ ratio becomes

$$\frac{[\mathrm{A}^-]}{[\mathrm{HA}]} = \frac{[\mathrm{HO}^{15}\mathrm{N}^{14}\mathrm{NO}^-] + [\mathrm{HO}^{14}\mathrm{N}^{15}\mathrm{NO}^-]}{[\textit{cyclic}\text{-HONNOH}] + [\textit{sym}\text{-HONNOH}]}$$

$$= \frac{2 \times 10^{\mathrm{pH}-\mathrm{p}K_\mathrm{a}}}{2 + \left(\frac{[\textit{sym}\text{-HONNOH}]}{[\textit{cyclic}\text{-HONNOH}]/2}\right)} = \frac{10^{\mathrm{pH}-\mathrm{p}K_\mathrm{a}}}{\left(\frac{2+K}{2}\right)} = 10^{\mathrm{pH}-\mathrm{p}K_{\mathrm{a,eff}}}, \tag{S19}$$

where $\mathrm{p}K_{\mathrm{a,eff}}$ is given by

$$\mathrm{p}K_{\mathrm{a,eff}} = \mathrm{p}K_a + \log\left(\frac{2+K}{2}\right). \tag{S20}$$

On the other hand, the above-mentioned $[\mathrm{A}^-]/[\mathrm{HA}]_{\mathrm{eff}}$ ratio can be expressed as:

$$\frac{[\mathrm{A}^-]}{[\mathrm{HA}]_{\mathrm{eff}}} = \frac{[\mathrm{HO}^{15}\mathrm{N}^{14}\mathrm{NO}^-] + [\mathrm{HO}^{14}\mathrm{N}^{15}\mathrm{NO}^-]}{[\textit{cyclic}\text{-HO}^{15}\mathrm{N}^{14}\mathrm{NOH}] + [\textit{cyclic}\text{-HO}^{14}\mathrm{N}^{15}\mathrm{NOH}]} = 10^{\mathrm{pH}-\mathrm{p}K_\mathrm{a}} \tag{S21}$$

Eqs. S19 to S21 show that $[\mathrm{HA}]_{\mathrm{eff}}$ and [HA] (or $\mathrm{p}K_\mathrm{a}$ and $\mathrm{p}K_{\mathrm{a,eff}}$) differ by a factor of (2+K)/2 (or by a value of log((2+K)/2)). In this work, with a free enthalpy difference of $\Delta G(\mathrm{sym} - \mathrm{cyclic}) = -2.6$ kJ/mol and $K = 2.9$, the correction term yields an effective acid constant shifted by +0.39. Note that inclusion of the symmetry factor 2 (which would be appropriate when ignoring the isotopic composition), with $K = 1.43$ and $\log{(1+K)/1} = 0.39$ would yield the same correction term.

Depending on the requested quantity, care needs to be taken to use the appropriate values. As outlined in Eqs. S15 and S16, $[\mathrm{HA}]_{\mathrm{eff}}$ and $\mathrm{p}K_\mathrm{a}$ determine the $\delta^{15}N^{\mathrm{SP}}_{\mathrm{total}}$ site preference instead of [HA] and $\mathrm{p}K_{\mathrm{a,eff}}$. However, as indicated by Eq. S8, rate constants in connection with the *sym*-HONNOH conformer of hyponitrous acid need to be accounted for when predicting the overall site preference of hyponitrous acid decomposition. Of course, assuming rapid equilibration, $\mathrm{EIE}_{\mathrm{HA}}$ can be calculated from $\Delta G(\mathbf{HO}^{15}\mathrm{N}^{14}\mathrm{N}\mathbf{OH}-\ \mathbf{HO}^{14}\mathrm{N}^{15}\mathrm{N}\mathbf{OH})$ as well.

(v) ^{15}N-site preference of $trans$-HONNO$^-$ decomposition

On the *trans* dimerization pathway, N_2O formation takes place by decomposition of *trans*-HONNO$^-$. This species is in acid-base equilibrium with *trans*-HONNOH, however, due to the absence of an H_2O elimation pathway, HONNOH can be considered a stable species. Figure S2 below summarizes the equilibria between nine isotopic species that have been considered for the derivation of an analytical expression for the total $\delta^{15}\mathrm{N}^{\mathrm{trans}}_{\mathrm{total}}$.

The conformers with the OH groups pointing outwards or inwards are connected by very fast rotations of the OH groups around the HO-N single bonds. At room temperature, the ratio of the *trans*-HONNO$^-$ with inwards- and outwards-pointing OH group, [Iso1]/[Iso2], is about 3/2 and the ratio of the corresponding decomposition rate constants, $k_{\mathrm{Iso1}}/k_{\mathrm{Iso2}}$, is about 1/12 (see Tables S5 and S6). Therefore, the overall decompositon is dominated by the decomposition of the slightly less abundant Iso2 species. Assuming that all the very fast rotational and fast acid-base equilibria always remain set, the calculation of the site preference can be based on the relative abundances of the four species Iso1α, Iso1β, Iso2α, and Iso2α, each weighted with the corresponding decomposition rate constant value.

The relative abundances (x) of the four species (in terms of a relative mole fraction) is given by the Boltzmann factors, with Z representing the state sum over all four relevant species:

$$x_i = \frac{\exp{(-G_i/RT)}}{Z} \quad \text{with} \quad Z = \sum_i \exp{(-G_i/RT)}. \tag{2S2}$$

Values are listed in Tables S7 and S8. The instantaneous formation of the α and β isotopmomers of N_2O can be written as

$$\frac{\mathrm{d}[^{14}\mathrm{N}^{15}\mathrm{NO}]}{\mathrm{d}t} \propto \left[\frac{x_{\mathrm{Iso1}\alpha}}{x_{\mathrm{Iso1}\alpha}+x_{\mathrm{Iso2}\alpha}} \times k_{\mathrm{Iso1}\alpha} + \frac{x_{\mathrm{Iso2}\alpha}}{x_{\mathrm{Iso1}\alpha}+x_{\mathrm{Iso2}\alpha}} \times k_{\mathrm{Iso2}\alpha}\right] \times (x_{\mathrm{Iso1}\alpha}+x_{\mathrm{Iso2}\alpha}) \tag{S23}$$

$$\frac{\mathrm{d}[^{15}\mathrm{N}^{14}\mathrm{NO}]}{\mathrm{d}t} \propto x_{\mathrm{Iso1}\beta} \times k_{\mathrm{Iso1}\beta} + x_{\mathrm{Iso2}\beta} \times k_{\mathrm{Iso2}\beta} \tag{S24}$$

This applies to the entire reaction process such that the ratio of α- and β-N_2O and with it the site preference can be calculated as follows:

$$\frac{[^{14}\mathrm{N}^{15}\mathrm{NO}]}{[^{15}\mathrm{N}^{14}\mathrm{NO}]} = \frac{x_{\mathrm{Iso1}\alpha} \times k_{\mathrm{Iso1}\alpha} + x_{\mathrm{Iso2}\alpha} \times k_{\mathrm{Iso2}\alpha}}{x_{\mathrm{Iso1}\beta} \times k_{\mathrm{Iso1}\beta} + x_{\mathrm{Iso2}\beta} \times k_{\mathrm{Iso2}\beta}} \tag{S25}$$

The resulting site preference values $\delta^{15}\mathrm{N}^{\mathrm{trans}}_{\mathrm{total}}$ (assuming no, Wigner, or Eckart tunneling) are listed in Table S8 and are included in Figures 5 and 6 in the main text.

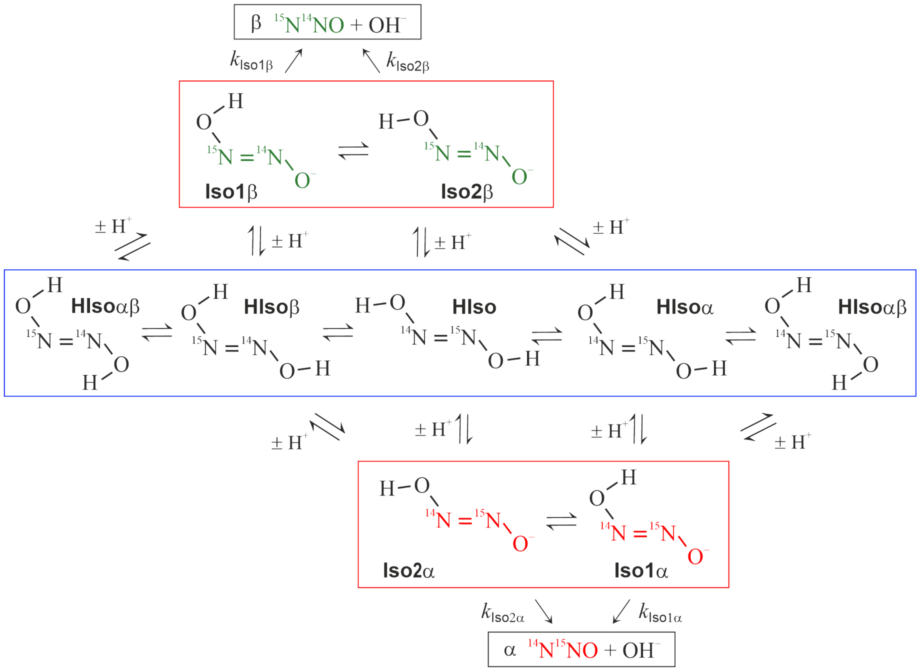

Figure S2: Reaction diagram for the *trans*-pathway of HNO dimerization.

C. Calculated Rate Constants at Selected Temperatures

Table S 1: Rate constants, $k(T)$, with and without tunneling factor, $\kappa(T)$, assuming one-dimensional Eckart type potential based on the B3LYP/aug-cc-PVTZ calculations for the dissociation and isomerization channels of *cis*-HO^{14}N^{15}NO^{-} intermediate (α-pathway) at selected temperatures in the range from 278 to 340 K.

T / K	Dissociation			Isomerization		
	$\kappa(T)$	$k_{\alpha,\mathrm{A}^-}(T)$ (10^7 s^{-1})		$\kappa(T)$	$k_{-1}(T)$ (10^{12} s^{-1})	
		a	b		a	b
278	1.14	0.5840	0.6654	3.39	0.3288	1.1155
297	1.12	1.6991	1.9049	3.06	0.4052	1.2392
316	1.11	4.3792	4.8450	2.80	0.4872	1.3627
336	1.09	10.637	11.633	2.58	0.5786	1.4921

[a] Rate constants without tunneling correction. [b] Rate constants with Eckart tunneling correction.

Table S 2: Rate constants, $k(T)$, with and without tunneling factor, $\kappa(T)$, assuming one-dimensional Eckart type potential based on the B3LYP/aug-cc-PVTZ calculations for the dissociation and isomerization channels of *cis*-HO^{15}N^{14}NO^{-} intermediate (β-pathway) at selected temperatures in the range from 278 to 340 K.

T / K	Dissociation			Isomerization		
	$\kappa(T)$	$k_{\beta,\mathrm{A}^-}(T)$ (10^7 s^{-1})		$\kappa(T)$	$k_{1}(T)$ (10^{12} s^{-1})	
		a	b		a	b
278	1.14	0.5652	0.6420	3.39	0.3321	1.1269
297	1.12	1.6479	1.8428	3.06	0.4089	1.2507
316	1.10	4.2552	4.6973	2.80	0.4913	1.3743
336	1.09	10.353	11.301	2.58	0.5831	1.5037

[a] Rate constants without tunneling correction. [b] Rate constants with Eckart tunneling correction.

Table S 3: Rate constants, $k(T)$, with and without tunneling factor, $\kappa(T)$, assuming one-dimensional Eckart-type potential based on B3LYP/aug-cc-PVTZ calculations for the dissociation and isomerization channels of *cis*-$HO^{14}N^{15}NOH$ intermediate (α-pathway) at selected temperatures from 278 to 340 K.

T / K	Dissociation			Isomerization				
	$\kappa(T)$	$k_{\alpha,\mathrm{HA}}(T)$ (10^2 s^{-1})		$\kappa(T)$	$k_2(T)$ (10^8 s^{-1})		$k_{-2}(T)$ (10^8 s^{-1})	
		a	b		a	b	a	b
278	12.5	0.1287	1.6045	1.31	0.2527	0.3299	0.7963	1.0397
297	8.07	0.7713	6.2271	1.26	0.6088	0.7692	1.7715	2.2383
316	5.84	3.7314	21.782	1.23	1.3214	1.6254	3.5840	4.4086
336	4.50	16.193	72.857	1.20	2.721	3.2700	6.912	8.3057

[a] Rate constants without tunneling correction. [b] Rate constants with Eckart tunneling correction.

Table S 4: Rate constants, $k(T)$, with and without tunneling factor, $\kappa(T)$, assuming one-dimensional Eckart type potential based on the B3LYP/aug-cc-PVTZ calculations for the dissociation and isomerization channels of *cis*-$HO^{15}N^{14}NOH$ intermediate (β-pathway) at selected temperatures in the range from 278 to 340 K.

T / K	Dissociation			Isomerization				
	$\kappa(T)$	$k_{\beta,\mathrm{HA}}(T)$ (10^2 s^{-1})		$\kappa(T)$	$k_3(T)$ (10^8 s^{-1})		$k_{-3}(T)$ (10^8 s^{-1})	
		a	b		a	b	a	b
278	12.4	0.1256	1.5517	1.31	0.8025	1.0480	0.2537	0.3313
297	8.01	0.7545	6.0458	1.26	1.7841	2.2547	0.6111	0.7723
316	5.80	3.6568	21.215	1.23	3.6075	4.4384	1.3261	1.6315
336	4.48	15.897	71.154	1.20	6.9538	8.3575	2.7303	3.2814

[a] Rate constants without tunneling correction. [b] Rate constants with Eckart tunneling correction.

D. trans-Pathway Data

Table S 5: Rate constants for the decomposition of the *trans*-$HONNO^-$ with the OH group pointing inward: $k_{\alpha,\mathrm{Iso1}}$ and $k_{\beta,\mathrm{Iso1}}$ at selected temperatures in the range from 278 to 340 K.

T / K	$k_{\mathrm{Iso1}\alpha}/\mathrm{s}^{-1}$			$k_{\mathrm{Iso1}\beta}/\mathrm{s}^{-1}$		
	a	b	c	a	b	c
278	0.09114	0.11655	0.12166	0.09230	0.11868	0.12415
297	0.81392	1.01319	1.04696	0.82399	1.03076	1.06687
316	5.61507	6.83191	7.00758	5.68278	6.94502	7.13279
336	34.0272	40.5634	41.3678	34.42778	41.2058	42.0654

[a] Without tunneling correction [b] With Wigner tunneling correction. [c] With Eckart tunneling correction.

Table S 6: Rate constants for the decomposition of the *trans*-$HONNO^-$ with the OH group pointing outward: $k_{\alpha,\mathrm{Iso2}}$ and $k_{\beta,\mathrm{Iso2}}$ at selected temperatures in the range from 278 to 340 K.

T / K	$k_{\mathrm{Iso2}\alpha}/\mathrm{s}^{-1}$			$k_{\mathrm{Iso2}\beta}/\mathrm{s}^{-1}$		
	a	b	c	a	b	c
278	1.08114	1.36766	1.42067	1.09318	1.39004	1.44663
297	7.94193	9.79008	10.07763	8.02897	9.94344	10.25038
316	46.1087	55.6080	56.8633	46.6070	56.4457	57.7855
336	237.857	281.300	286.173	240.394	285.384	290.586

[a] Without tunneling correction [b] With Wigner tunneling correction. [c] With Eckart tunneling correction.

Table S 7: Free enthalpies (ΔG) and relative free enthalpies ($\Delta\Delta G$) involved in the dissociation of α and β pathway of *trans*-HONNO$^-$ for both isomers 1 and 2 (in kJ/mol), see Figure S2.

Species	Label	ΔG (kJ/mol)	$\Delta\Delta G$ (kJ/mol)
trans-HO^{14}N^{15}N^{16}O	Iso1α	-684423.78623	0.00000
trans-HO^{15}N^{14}N^{16}O	Iso1β	-684423.75735	0.02888
TS1	Iso1α	-684350.63193	73.15431
TS2	Iso1β	-684350.63455	73.15168
trans-HO^{14}N^{15}N^{16}O	Iso2α	-684422.73341	1.05283
trans-HO^{15}N^{14}N^{16}O	Iso2β	-684422.70978	1.07645
TS1	Iso2α	-684355.21605	68.57018
TS2	Iso2β	-684355.22130	68.56493
^{14}N^{15}N^{16}O + OH	Iso2α	-684525.89455	-102.10832
^{15}N^{14}N^{16}O + OH	Iso2β	-684525.78166	-101.99542

Table S 8: Relative mole fraction, x_i, of the various trans-HONNO$^-$ isomers, given by the Boltzmann factor with the state sum Z, as well as the resulting total site preference $\delta^{15}\text{N}^{\text{trans}}_{\text{total}}$ from Eq. S25 for the *trans*-HONNO$^-$ decomposition at selected temperatures.

T / K		278 K	297 K	316 K	336 K
Z		3.2494	3.2879	3.3228	3.3560
$x_{\text{Iso1}\alpha}$		0.3077	0.3041	0.3010	0.2980
$x_{\text{Iso1}\beta}$		0.3039	0.3006	0.2977	0.2949
$x_{\text{Iso2}\alpha}$		0.1952	0.1986	0.2016	0.2044
$x_{\text{Iso2}\beta}$		0.1932	0.1967	0.1998	0.2027
$\delta^{15}\text{N}^{\text{trans}}_{\text{total}}$ / ‰	no tunneling	-0.77	-1.23	-1.64	-2.02
$\delta^{15}\text{N}^{\text{trans}}_{\text{total}}$ / ‰	Wigner tunneling	-5.94	-5.89	-5.86	-5.84
$\delta^{15}\text{N}^{\text{trans}}_{\text{total}}$ / ‰	Eckart tunneling	-7.84	-7.36	-7.01	-6.74
$\text{EIE}_{\text{effective}}$	Eckart tunneling	10.59	9.96	9.41	8.89
$\text{KIE}_{\text{effective}}$	Eckart tunneling	-18.23	-17.14	-16.25	-15.5

E. Basis Set Dependence

Table S 9: Basis set dependence of the calculated (ISOEFF98, without tunneling) intrinsic isotope effects for *cis*-hyponitrite anion and cis-hyponitrous acid decomposition at $T = 297$ K.

	$\delta^{15}N^{SP}_{KIE,HA}$ / ‰	$\delta^{15}N^{SP}_{EIE,HA}$ / ‰	$\delta^{15}N^{SP}_{KIE,A^-}$ / ‰	$\delta^{15}N^{SP}_{EIE,A^-}$ / ‰
6-311+G(d,p)	23.2	3.1	30.3	10.1
6-311++G(d,p)	23.2	3.0	30.3	10.1
6-311++G(df,pd)	23.2	3.1	31.6	9.5
aug-cc-PVDZ	22.3	3.0	32.1	9.5
aug-cc-PVTZ	22.7	3.1	31.0	9.6
aug-cc-PVQZ	22.6	3.1	31.4	9.6

Table S 10: Basis set dependence of electronic energies (offset by +260 Hartree, without ZPE correction) for selected transition states and reactant complexes (see Figures 2 and 3 in main text).

	cis-anion intermediate			*cis*-neutral intermediate			
	TS1	TS2/TS3	RC1/RC2	TS1'/TS2'	TS3'/TS4'	RC1'	RC2'/RC3'
6-311+G(d,p)	-0.665249	-0.656276	-0.671516	-1.113971	-1.095148	-1.127234	-1.125207
6-311++G(d,p)	-0.665287	-0.656325	-0.671578	-1.114083	-1.095277	-1.127329	-1.125310
6-311++G(df,pd)	-0.672742	-0.662642	-0.678183	-1.121802	-1.103315	-1.135120	-1.133050
aug-cc-PVDZ	-0.630511	-0.616822	-0.635954	-1.077946	-1.059905	-1.091250	-1.089691
aug-cc-PVTZ	-0.688189	-0.677688	-0.693867	-1.138923	-1.121242	-1.151890	-1.150339
aug-cc-PVQZ	-0.706607	-0.696023	-0.712387	-1.157924	-1.139848	-1.170977	-1.169452

Table S 11: Basis set dependence of imaginary frequencies (in cm^{-1}) of selected transition states (see Figures 2 and 3 in main text).

	cis-anion intermediate		*cis*-neutral intermediate			
	TS1	TS2	TS1'	TS2'	TS3'	TS4'
6-311+G(d,p)	-1216.767	-302.625	-483.374	-1318.001	-483.597	-1316.340
6-311++G(d,p)	-1217.393	-303.289	-483.278	-1317.118	-483.054	-1315.415
6-311++G(df,pd)	-1181.621	-319.798	-485.316	-1268.093	-485.549	-1266.145
aug-cc-PVDZ	-1197.800	-312.174	-473.163	-1184.315	-473.384	-1182.353
aug-cc-PVTZ	-1190.979	-329.644	-467.084	-1257.809	-467.306	-1257.033
aug-cc-PVQZ	-1199.366	-329.704	-469.746	-1268.866	-469.858	-1266.980

F. Selected Line Pairs from Comb Spectroscopy

Table S 12: Selected transitions for retrieving $\delta^{15}N^{SP}$ and the difference in the lower state term values ($\Delta E = E_{\alpha''} - E_{\beta''}$) of the two isotopomers.

Transition	ΔE / cm^{-1}	Transition	ΔE /cm^{-1}	Transition	ΔE / cm^{-1}
P(31)	14.0	P(12)	2.20	R(11)	1.86
P(30)	13.1	P(11)	1.86	R(12)	2.20
P(28)	11.5	P(10)	1.55	R(14)	2.97
P(24)	8.50	P(9)	1.27	R(15)	3.39
P(22)	7.14	P(6)	0.59	R(19)	5.37
P(20)	5.93	P(5)	0.42	R(25)	9.18
P(19)	5.37	P(4)	0.28	R(26)	9.91
P(17)	4.32	R(3)	0.17	R(27)	10.7
P(15)	3.39	R(4)	0.28	R(28)	11.5
P(31)	14.0	R(10)	1.55	R(29)	12.3

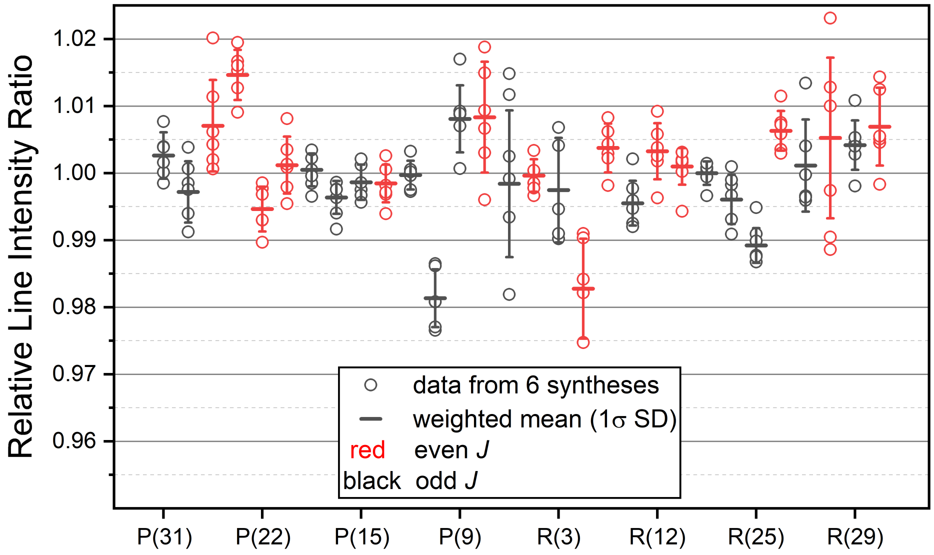

Figure S 3: Statistics of fitted relative line intensity ratios (with respect to HITRAN2020 line strength data) from 6 different N_2O syntheses for the 30 selected N_2O lines (see Table S 12). The horizontal dashes represent the weighted means with the error bars corresponding to the 1σ standard deviations of the six data points for each single line pair ratio. Black color corresponds to lines with odd rotational quantum number J and red color to even J. To allow for a direct comparison of the data from six different syntheses (that correspond to variable $\delta^{15}N^{SP}$ value), the line pair intensity ratios for each single spectrum have been normalized to its mean value. Note that the repeatability of the intensity measurements of the single line pairs (in average $\pm 0.37\%$) is significantly better than the overall scatter of the data points with a 1σ weighted standard deviation of ($\pm 0.76\%$). We attribute the clearly visible systematic over- and underestimation of several line intensities as an uncertainty arising from the line intensity ratios extracted from the HITRAN2020 database. Note that the resulting $\delta^{15}N^{SP}$ corresponds to the mean of all values such that corresponding standard error of the mean is reduced by a factor of $\sqrt{30}$, hence resulting in uncertainties of $\pm 0.68‰$ and $\pm 1.4‰$ in $\delta^{15}N^{SP}$, respectively.
